# Environmental drivers of broiler carcass condemnation in humid subtropical regions: an exploratory study on the association of lagged climatic effects

**DOI:** 10.1007/s11250-026-05081-y

**Published:** 2026-05-20

**Authors:** Alessandro Silva Lopes, Guilherme Francisco Sobierai Batista, Denise Ortigosa Stolf, Marcel Manente Boiago, Alessandro Cazonatto Galvão, Weber da Silva Robazza

**Affiliations:** 1 Federal Inspection Service (SIF), Food Supply (MAPA), Ministry of Agriculture, Nova Erechim, Livestock, Santa Catarina, Brazil; 2 Department of Food and Chemical Engineering, Santa Catarina State University, Pinhalzinho, Santa Catarina Brazil; 3 UCEFF (Unidade Central de Educação FAEM Faculdade), Chapecó, Santa Catarina Brazil; 4 Department of Animal Science, Santa Catarina State University, Chapecó, Santa Catarina Brazil

**Keywords:** Carcass condemnation, Broiler production, Climatic variability, Lagged effects, Poultry health

## Abstract

Intensive poultry production in humid subtropical agro-ecologies faces unique environmental challenges that compromise carcass quality, welfare, and food security. While climatic influences are recognized, the temporal dimensions of these effects remain largely underexplored. This study quantified the immediate and time-lagged (1–3 months) influences of climatic variables on 12 causes of broiler carcass condemnation in a representative subtropical hub. Longitudinal data (2021–2023) from federally inspected slaughterhouses in Santa Catarina, Brazil, a relevant case study for poultry production within Köppen Cfa and Cfb climate zones, were analyzed using Spearman rank correlation and robust regression models, including Generalized Linear Models (GLM) with a quasibinomial distribution. Total condemnation rates rose from 13.1% in 2021 to 15.5% in 2023, dominated by gastrointestinal contamination (5.0%) and skin lesions (3.5%). Regression analysis revealed that lagged precipitation was a significant predictor for arthritis and cellulitis (*p* < 0.05), whereas higher temperatures and heat index reduced rejections for ascites and skin lesions (*p* < 0.01). A quasibinomial GLM identified lagged heat index (β = − 0.096, *p* = 0.051) as a marginal predictor for inflammatory lesions. These findings suggest an association between environmental conditions during the initial stages of rearing and the final carcass quality. The established models offer insights for seasonal management across similar humid subtropical belts to mitigate economic losses and enhance production sustainability.

**Supplementary Information:**

The online version contains supplementary material available at 10.1007/s11250-026-05081-y.

## Introduction

The global intensification of the poultry industry has increasingly centered on tropical and subtropical regions, which now account for a significant portion of the world’s animal protein supply (Alvares et al. 2013; Chaiban et al. 2020). In these agro-ecological zones, particularly those characterized by humid subtropical climates (Köppen-Geiger Cfa and Cfb), intensive broiler production faces unique environmental challenges (Liu et al. 2020). High thermal amplitude, seasonal surges in precipitation, and sustained relative humidity above 70% are primary stressors that mediate host-pathogen interactions and metabolic homeostasis (Mottet and Tempio 2017).

The efficiency and sustainability of these systems are measured by their ability to deliver wholesome carcasses. Any deviation from clinical health or processing standards results in carcass condemnation, the total or partial rejection of meat during sanitary inspection. Beyond the immediate economic impact, which can represent substantial annual losses for individual processing facilities (Hortêncio et al. 2022), condemnations represent a significant source of inefficiency and food loss in the food value chain (Buzdugan et al. 2020). This food waste directly undermines the United Nations’ Sustainable Development Goals (SDGs), specifically Zero Hunger (SDG 2) and Responsible Consumption and Production (SDG 12), while signaling compromised animal welfare on the farm (Karlsson et al. 2025).

In humid subtropical belts, encompassing production hubs in Southern Brazil, the Southeastern United States, Southern China, and Eastern Australia, carcass rejections often follow cyclical patterns (Meteyake et al. 2020; Kpomasse et al. 2021). Pathologies such as ascites syndrome are strongly linked to environmental stressors during specific seasonal windows (Hu and Cheng 2021), whereas inflammatory lesions, cellulitis, and arthritis are mediated by litter moisture and ambient humidity (Wideman et al. 2013; Pirompud et al. 2023). However, the biological impact of environmental stressors is rarely instantaneous. Delayed physiological responses or the gradual deterioration of litter quality suggest that climatic conditions weeks or months prior to slaughter may be more predictive of condemnation rates than concurrent weather on the day of processing (Kang et al. 2020; Nawaz et al. 2021).

Despite the global significance of these subtropical belts, comprehensive models quantifying lagged meteorological effects on condemnation causes remain scarce. Most existing literature focuses on single-cause analyses or concurrent climatic effects, failing to capture the complex temporal dynamics of intensive avian health in these sensitive environments (Hortêncio et al. 2022; Carvalho et al. 2025; Pirompud et al. 2024). Furthermore, while recent studies have employed Machine Learning (ML) for predictive accuracy, these ‘black-box’ models often lack biological interpretability. This study fills this gap by utilizing a multi-cause approach with a 1–3 month lag structure, prioritizing the mechanistic understanding of how environmental stressors influence specific poultry pathologies over successive production cycles.

Santa Catarina, Brazil, serves as an ideal case study for this research due to its status as a premier regional poultry exporter operating high-density intensive systems under representative Cfa/Cfb climates. Therefore, quantifying the environmental drivers of poultry health in this region provides a regional framework that may inform similar production systems across the humid subtropical belt (Liu et al. 2020).

This study aims to quantify the effects of temperature, precipitation, humidity, and heat index, with time lags of 1 to 3 months, on the condemnation rates for 12 distinct causes in federally inspected broiler slaughterhouses between 2021 and 2023. By utilizing robust regression techniques, including Generalized Linear Models with a quasibinomial distribution, this research seeks to establish a predictive environmental framework to mitigate economic losses and enhance animal welfare in subtropical agro-ecologies.

## Materials and methods

### Study site and representative subtropical context

The study was conducted using data from broiler production systems in the West region of Santa Catarina, Southern Brazil. This region is a relevant regional case study for intensive poultry production, characterized by a humid subtropical climate, Köppen-Geiger types Cfa and Cfb (Alvares et al. 2013). This agro-ecological zone is characterized by well-distributed rainfall throughout the year and pronounced thermal amplitude, mirroring intensive poultry hubs in the Southeastern United States, Southern China, and Eastern Australia. The municipality of Chapecó serves as the primary processing hub for this region, which accounts for approximately 79.4% of the state’s poultry output.

### Carcass condemnation data and sanitary inspection

Data on carcass condemnations were retrieved from the management information system of the Federal Inspection Service (SIF), under Brazil’s Ministry of Agriculture, Livestock, and Food Supply (MAPA), covering the period from February 2021 to December 2023 (MAPA 2017). The records were filtered for broilers, excluding animals that died during transport or pre-slaughter phases. Twelve primary condemnation causes were analyzed: gastrointestinal contamination (GC), skin lesions (SL), arthritis (AR), airsacculitis (AI), cellulitis (CE), processing failures (PF), traumatic lesions (TL), ascites (AS), visual defects (VD), septicaemia (SE), cachexia (CA), and inflammatory lesions (IL) (Salines et al. 2017). Partial and total condemnations were merged into a single variable for each cause to standardize the quantitative analysis. As the primary objective was to identify environmental drivers rather than assessing slaughterhouse yield, this aggregation was adopted to capture the overall prevalence of each pathology, regardless of the severity of the lesions at the time of inspection, following methodologies used in similar regional epidemiological studies (Belintani et al. 2019; Muchon et al. 2019).

### Environmental monitoring and climate indices

Meteorological data were obtained from the Chapecó climate station, maintained by the National Institute of Meteorology (INMET). The variables included total monthly precipitation (mm), mean monthly temperature (°C), and mean relative humidity (%). To quantify the combined effect of temperature and moisture, the Heat Index (HI) was calculated using Eq. 1, where 𝑇 is the mean monthly temperature (°F), and 𝑅𝐻 is the mean monthly relative humidity (%) (Steadman 1979). Although originally developed for human physiology, this index has been successfully used as a proxy for environmental thermal stress in poultry production studies in tropical and subtropical regions where specific avian indices may lack standardized long-term meteorological station data (Kang et al. 2020). While specific avian bioclimatic indices exist, the Heat Index (HI) provides a robust and comparable metric when utilizing standardized long-term data from public meteorological stations, which frequently lack the specific variables required for more complex avian-specific formulas.

1 $$\eqalign{& HI = - 42.379 + 2.049T + 10.143RH - 0.225T \cr & \,\,\,\,\,\,\,\,\,\,\,\,\,\,\, \times \>RH - 6.838 \times \>{10^{ - 3}}{T^2} - 5.482 \times \>{10^{ - 2}}R{H^2} \cr & \,\,\,\,\,\,\,\,\,\,\,\,\,\,\,\,\,\, + 1.229 \times \>{10^{ - 3}}{T^2} \times \>RH + 8.528 \times \>{10^{ - 4}}T \cr & \,\,\,\,\,\,\,\,\,\,\,\,\,\,\,\,\,\,\,\,\,\,\,\,\, \times \>R{H^2} - 1.99 \times \>{10^{ - 6}}{T^2} \times \>R{H^2} \cr}$$

### Statistical modeling and exploratory analysis

Monthly aggregated data (Feb 2021-Dec 2023; *n* = 34 observations) were analyzed. This temporal resolution was chosen to align climatic variables with the standardized monthly sanitary reports provided by federal authorities. While monthly aggregation may smooth acute, short-term climatic peaks, it is appropriate for identifying broader seasonal trends and lagged environmental effects that influence flock-level health outcomes (Kang et al. 2020). Given the limited number of time points relative to the potential predictors, modeling strategies prioritized parsimony and biological plausibility. To explore the nonlinear relationship between climatic drivers and poultry health, Spearman rank correlation (ρ) was applied to concurrent data and time-lagged variables (1, 2, and 3 months). Spearman’s rank correlation was chosen because the datasets are nonlinear and nonnormal (Zar 2010). For each month, the total values for each condemnation cause were divided by the total number of slaughters in that month and multiplied by 100 to obtain percentage values, ensuring that correlations reflected condemnation proportions rather than total numbers. Descriptive statistics regarding the data obtained are reported in Tables S1 (condemnation causes) and S2 (meteorological variables). Additionally, because the effects of climatic variables on various aspects of poultry production may not be immediate (Kumar et al. 2021), heatmaps accounting for time lags of 1, 2, and 3 months were produced. Correlation coefficients with p-values less than 0.05 were considered statistically significant. All heatmaps were created using the R package corrplot (Wei and Simko 2024).

To quantify the effects of climatic variables on the total condemnation rate and each of the 12 condemnation causes, as dependent variables, multiple linear regression models were initially fitted using ordinary least squares (OLS) in R version 4.3.3 (R Core Team 2024). The dependent variable $\:Y$, representing the condemnation rate for each cause, was modeled (Eq. 2) as a function of the concurrent and lagged (1-month) climatic variables: precipitation (mm, $\:{X}_{1}$), mean temperature (°C, $\:{X}_{2}$), mean humidity (%, $\:{X}_{3}$), heat index (a composite measure of temperature and humidity, $\:{X}_{4}$), the precipitation lag-1 (mm, $\:{X}_{5}$), mean temperature lag-1 ($\:{X}_{6}$, °C), mean humidity lag-1 (%, $\:{X}_{7}$), and heat index lag-1 ($\:{X}_{8}$). These variables were selected based on their relevance to slaughterhouse condemnations and prior Spearman correlation analyses. The maximum and minimum temperatures and humidities were excluded because they were highly correlated with their respective means, thereby reducing multicollinearity. Month and year were included as categorical control variables to account for seasonality and temporal trends, respectively. April was selected as the reference month because it is a transition season, with intermediate climatic conditions, and 2021 was selected as the reference year because it was the first year of analysis.

2 $$\>Y = \beta {\>_0} + \sum {\>_{{\rm{i}} = 1}^8} \beta {\>_{\rm{i}}}{X_{\rm{i}}} + \sum {\>_{{\rm{i}} = 1}^{11}} \alpha {\>_{\rm{i}}}{\rm{Mont}}{{\rm{h}}_{\rm{i}}} + \sum {\>_{{\rm{i}} = 1}^2} \gamma {\>_{\rm{i}}}{\rm{Yea}}{{\rm{r}}_{\rm{i}}} + {\rm{\epsilon }}$$

where $\:{\beta\:}_{0}$ is the intercept, $\:{\beta\:}_{\mathrm{i}}$ (i = 1 to 8) are coefficients for the climatic variables, $\:{\alpha\:}_{\mathrm{i}}$ (i = 1 to 11) are coefficients for the month levels, $\:{\gamma\:}_{\mathrm{i}}$ (i = 1 to 2) are coefficients for the year levels, and $\:ϵ$ is the residual error.

To optimize model fit and select the most relevant predictors, stepwise regression was performed using the Akaike Information Criterion (AIC) with the stepAIC function from the MASS package (Venables and Ripley 2022). Predictors with *p* < 0.05 were considered significant, while those with *p* < 0.1 were reported as marginally significant to capture potential trends relevant to slaughterhouse conditions. Model diagnostics included tests for normality of residuals (Shapiro-Wilk test), homoscedasticity (Breusch-Pagan test, using the lmtest package; Zeileis and Hothorn 2022), and multicollinearity (Variance Inflation Factor, VIF, using the car package; Fox and Weisberg 2019). Complete diagnostic results, including Shapiro-Wilk p-values, Breusch-Pagan statistics, and full VIF tables for all 13 models (TC + 12 causes), are reported in Tables S3-S8. Moderate multicollinearity was observed in some stepwise models (e.g., VIF > 5 for $\:{X}_{2}$ up to 22.92 in the total condemnation (TC) model; GVIF^(1/(2df)^ > 4.5 in several; see Tables S3-S6). To address this, a robustness check using ridge regression (Zhang and Politis 2022) confirmed consistent effect directions and significance for key predictors. Predictors with high VIFs were retained because of their biological relevance to poultry health, such as interactions between temperature and humidity (Nawab et al. 2018). Post hoc power analysis (G*Power 3.1, Faul et al. 2007) confirmed > 80% power for key predictors (e.g., lagged precipitation in arthritis (AR)/cellulitis (CE) models: *n* = 34, effect size f^2^ = 0.15, α = 0.05).

For inflammatory lesions, the linear model violated assumptions (Shapiro-Wilk *p* = 0.0769, Breusch-Pagan *p* = 0.001, adjusted R^2^ = − 0.471 in the base model), leading to the adoption of a generalized linear model (GLM) with quasibinomial distribution. The GLM included the heat index, lag-1 heat index, and year. To ensure model adequacy, the dispersion parameter ($\:\phi\:$) was formally estimated to account for overdispersion, and zero inflation was assessed using score tests, proving non-significant for this pathology. Results were summarized using regression coefficients (β), standard errors, p-values, adjusted R^2^ for linear models, or deviance reduction and dispersion parameters for the GLM. Detailed regression tables for all models are provided in Supplementary Tables (S3-S6).

To ensure the validity of the time-series analysis, the Durbin-Watson test was performed to assess potential temporal autocorrelation in the residuals. Furthermore, given the sample size (*n* = 34), model selection prioritized parsimony to mitigate the risk of overfitting. Although some predictors exhibited multicollinearity (VIF > 5), the consistency observed between the Stepwise OLS results and the Ridge Regression coefficients confirms the reliability of the direction and significance of the identified associations. This cross-validation approach across different modeling techniques (OLS, Ridge, and GLM) was adopted to mitigate the risk of Type I errors (false positives) inherent in multiple comparisons, ensuring that only robust associations were prioritized instead of relying solely on adjusted p-values.

## Results

### Condemnation patterns in the representative subtropical hub

**Fig. 1:**
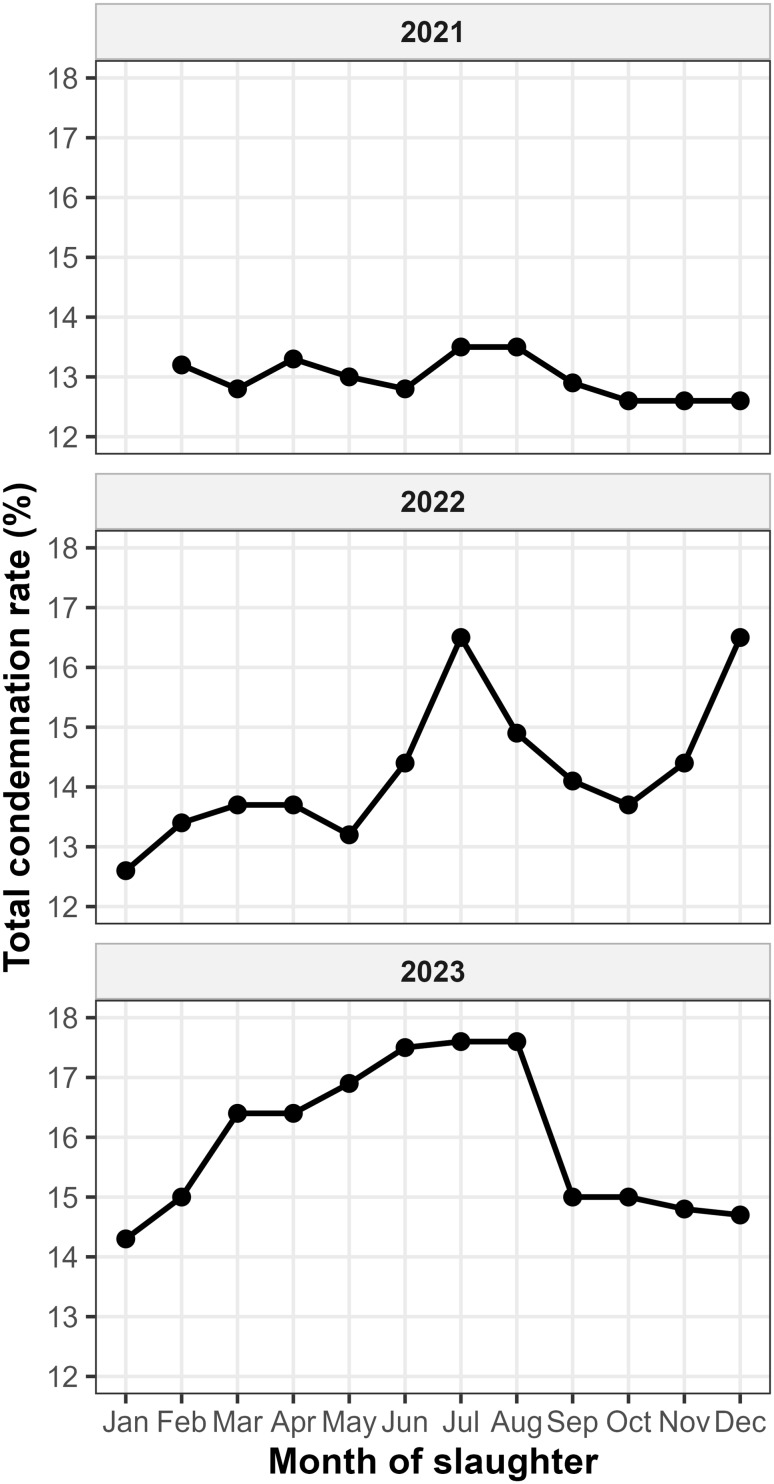
Monthly evolution of total broiler carcass condemnation rates in Santa Catarina, Brazil (2021–2023). The faceted panels illustrate the transition from a relatively stable pattern in 2021 to more pronounced seasonal fluctuations in 2022 and 2023. Noteworthy peaks are observed consistently during the late autumn and early winter window (May–July), aligning with adverse environmental conditions in subtropical humid zones. Data for 2021 begins in February

Between February 2021 and December 2023, the total condemnation rate (TC) in the intensive production hub of Santa Catarina, serving as a sentinel for humid subtropical regions, increased over the study period, rising from an average of 13.1% in 2021 to 15.5% in 2023. The temporal evolution of these rates is illustrated in Fig. 1. The data reveal a relatively stable pattern in 2021 with low variability, transitioning to a more complex seasonal profile in 2022 and 2023. In these latter years, a bimodal-like distribution is observed, with primary seasonal peaks consistently appearing within the May–July window, followed by a secondary elevation towards the end of 2022, while 2023 maintained consistently higher baseline rates throughout the second semester compared to 2021 followed by a secondary elevation towards the end of the year in 2022, while 2023 maintained consistently higher baseline rates throughout the second semester compared to previous years.

**Fig. 2:**
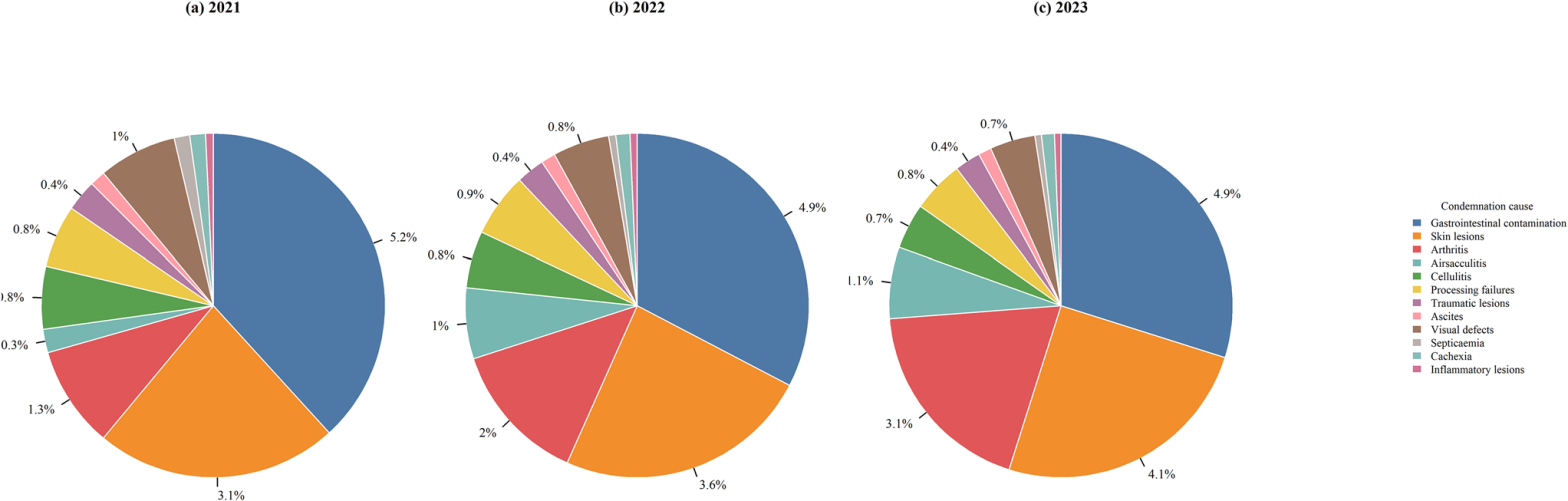
Proportion of condemnation causes in Santa Catarina relative to total animals slaughtered in poultry processing (**a**) 2021, (**b**) 2022, and (**c**) 2023

Analysis of the 12 primary causes (Fig. 2) revealed that gastrointestinal contamination (GC, 5.0%) and skin lesions (SL, 3.5%) were the dominant drivers of rejection. Other significant contributors included arthritis (AR, 1.3–2.3%), airsacculitis (AI, 0.2–1.4%), processing failures (PF, 0.7–0.9%), and cellulitis (CE, ~ 0.7%). Contamination and skin-related rejections remain the leading causes of rejection, consistent with findings from other high-density production hubs in Southern Brazil (Kanabata et al. 2023; Muchon et al. 2019). Descriptive statistics for annual production and condemnation rates are summarized in Table 1.

**Table 1:** Descriptive statistics for selected condemnation causes and the number of slaughters

Variable	Year	Mean	Standard Deviation	Minimum	Maximum
Animals Slaughtered (Million)	2021	44.07	2.87	41.97	50.86
	2022	48.18	7.79	38.77	58.86
	2023	61.03	3.94	55.68	68.06
Condemnation Rate	2021	0.1309	0.0051	0.1259	0.1424
	2022	0.1447	0.0129	0.1263	0.1647
	2023	0.1545	0.0137	0.1367	0.1760
Gastrointestinal Contamination Proportion	2021	0.0505	0.0020	0.0477	0.0535
	2022	0.0468	0.0040	0.0413	0.0527
	2023	0.0501	0.0042	0.0432	0.0548
Skin Lesions Proportion	2021	0.0326	0.0122	0.0204	0.0423
	2022	0.0352	0.0077	0.0289	0.0453
	2023	0.0376	0.0065	0.0293	0.0505
Arthritis Proportion	2021	0.0131	0.0026	0.0100	0.0186
	2022	0.0176	0.0040	0.0131	0.0236
	2023	0.0225	0.0083	0.0137	0.0370

### Environmental dynamics and time-lagged correlations

**Fig. 3:**
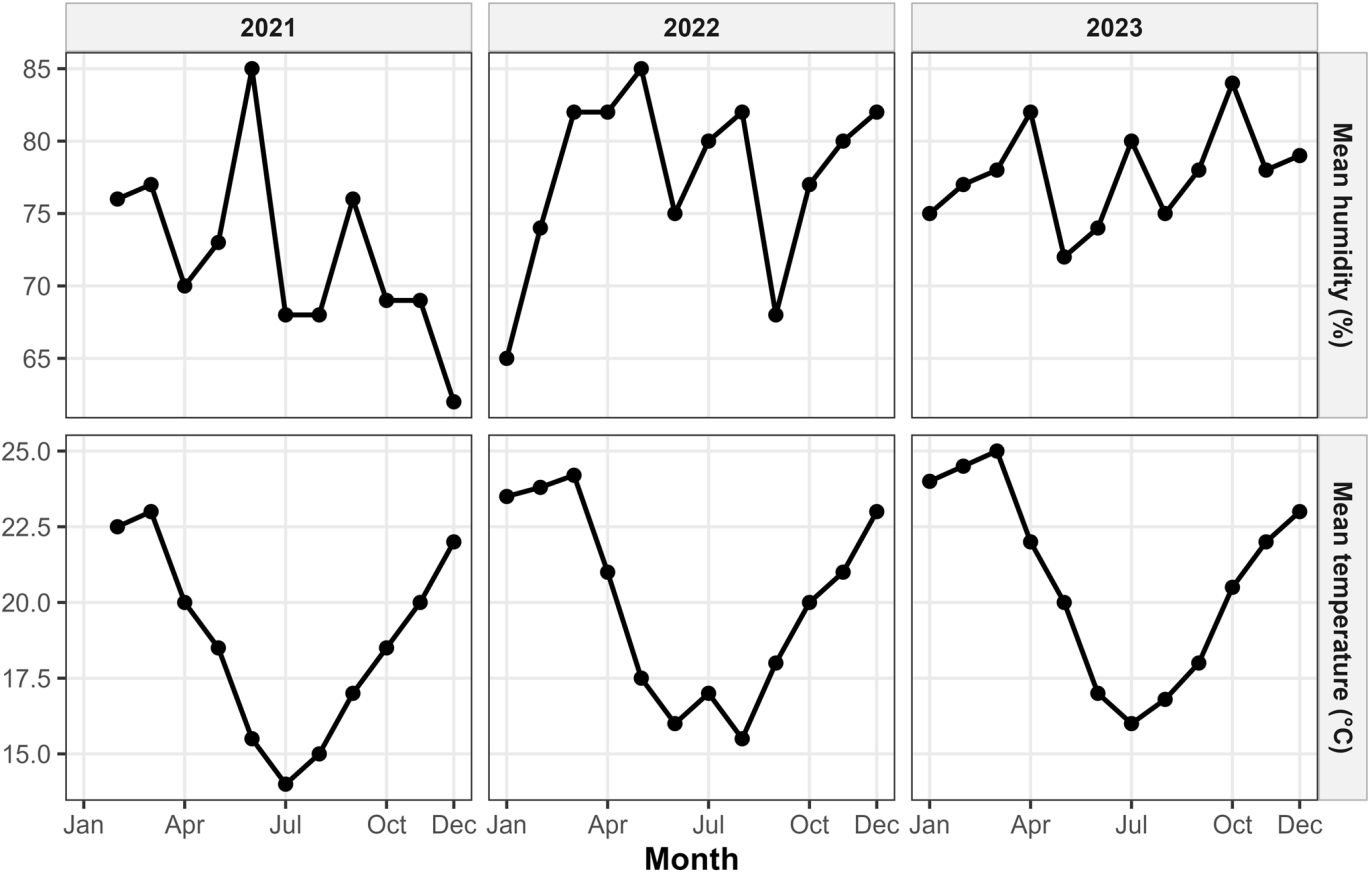
Time series plots of mean temperature (°C) and mean humidity (%) in Chapecó from 2021 to 2023

**Fig. 4:**
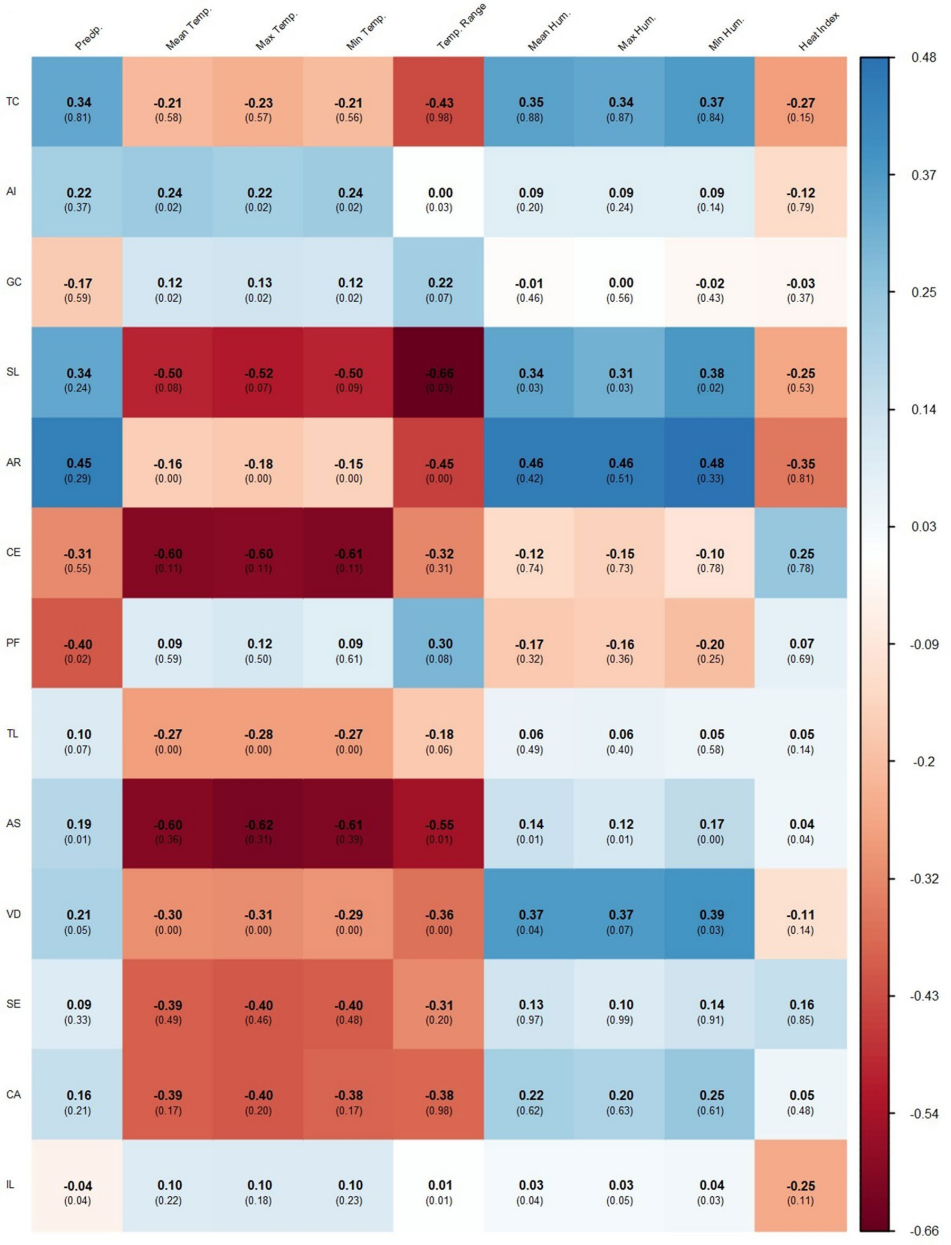
Correlation heatmap between poultry condemnation causes and climatic variables. Each cell displays the Spearman rank correlation coefficient and its corresponding p-value. P-values < 0.05 indicate statistically significant correlation. Note: GC: Gastrointestinal Contamination; SL: Skin Lesions; AR: Arthritis; AI: Airsacculitis; CE: Cellulitis; PF: Processing Failures; TL: Traumatic Lesions; AS: Ascites; VD: Visual Defects; SE: Septicaemia; CA: Cachexia; IL: Inflammatory Lesions

The behavior of climatic variables during the study period, including significant surges in precipitation and high relative humidity, is displayed in time-series plots (Fig. 3). Spearman rank correlation analysis (Fig. 4) identified that the influence of the subtropical environment on poultry health is often characterized by significant time lags. While concurrent temperature showed a weak positive correlation with TC (ρ = 0.24), lagged relative humidity at 2 months exhibited a much stronger positive association (ρ = 0.52, *p* < 0.05), as shown in the supplementary heatmaps (Figs. S1-S3).

Pathology-specific correlations further highlighted the role of seasonal stressors in Cfa climates:

- Airsacculitis (AI): Positively correlated with mean, maximum, and minimum temperatures (ρ = 0.22–0.24, *p* = 0.02) without lag (Fig. 4) and at a 1-month lag (Fig. S1).
- Ascites (AS): Strongly associated with winter conditions, showing a negative correlation with temperature range without lag (ρ = − 0.55, *p* = 0.01; Fig. 4).
- Arthritis (AR) and Cellulitis (CE): Demonstrated delayed responses to environmental moisture, with AR showing positive correlations with precipitation and humidity at 1 to 3-month lags (Figs. S1-S3).

### Predictive modeling of climatic stressors

Stepwise multiple linear regression and Generalized Linear Models (GLM) quantified the magnitude of environmental drivers while controlling for temporal effects (see results in Tables S4-S6). To validate these models, diagnostic tests were performed for each regression equation. Model diagnostics confirmed the absence of significant temporal autocorrelation in the residuals, with Durbin-Watson (DW) statistics ranging from 1.72 to 2.04 (*p* > 0.05) for the primary condemnation causes (detailed in Tables S3-S5). Additionally, ridge regression analysis was conducted as a robustness check for models with moderate collinearity (VIF > 5). The ridge coefficients yielded consistent effect directions and significance for all key predictors, reinforcing the stability of the stepwise selection results.

The model for Ascites (AS) demonstrated exceptional robustness (adjusted R^2^ = 0.975), driven by the negative effects of mean temperature (β = − 0.021) and heat index (β = − 0.008), confirming the cold-stress axis typical of subtropical winters. For inflammatory lesions (IL), the quasibinomial GLM successfully addressed overdispersion and heteroscedasticity, identifying the lagged heat index (β = − 0.096) as a significant environmental predictor. This suggests that warmer conditions in the month prior to slaughter are associated with a lower incidence of immune-mediated pathologies.

## Discussion

### The subtropical paradigm and environmental dynamics

The observed total condemnation rates in this study are higher than those reported for previous decades in Brazilian industrial poultry clusters (Muchon et al. 2019), reflecting the cumulative impact of production intensification and the evolving sensitivity of Federal Inspection Service (SIF) protocols. The increase in broiler carcass condemnation rates from 13.1% in 2021 to 15.5% in 2023 underscores a growing challenge for intensive poultry systems in humid subtropical regions (Kpomasse et al. 2021). The transition to a bimodal distribution in 2022 and 2023 (Fig. 1), with consistent peaks in the May-June window, identifies a seasonal vulnerability tied to the climatic characteristics of Köppen-Geiger Cfa and Cfb zones (Alvares et al. 2013). These agro-ecological zones, characterized by high relative humidity and significant thermal amplitude, mirror production conditions in the Southeastern United States, Southern China, and Eastern Australia, making these findings potentially relevant for other intensive systems in these regions (Peel et al. 2007; Thobe et al. 2025).

While global broiler condemnation rates are often reported around 1.04% for total rejection in some regions, the high rates observed here (reflecting the sum of partial and total rejections) highlight the sensitivity of high-performance genetic strains to environmental stressors in the subtropics (Salines et al. 2017; Belintani et al. 2019). The significant “year effects” identified in the regression models for 2022 and 2023 suggest that, beyond climate, operational factors such as refined inspection protocols or changes in slaughter-line technology may be associated with the data trends.

### Delayed environmental effects on health causes

A novel contribution of this study is the identification of the temporal dimension of poultry rejections. Gastrointestinal contamination (GC, 5.0%), the leading cause of rejection, exhibited strengthened correlations with temperature at 2- and 3-month lags (ρ = 0.62–0.63, *p* = 0.02; Fig. S2). Unlike previous studies focusing on slaughter-day weather, this suggests that environmental conditions during the early rearing phases are potentially linked to the microbial load or intestinal integrity of the birds at the end of the cycle (Rouger et al. 2017).

For inflammatory lesions (IL), the use of a Generalized Linear Model (GLM) with a quasibinomial distribution was essential to handle the overdispersion of proportional data (Table S6). The identification of the lagged heat index (β = − 0.096, *p* = 0.051) as a marginal predictor suggests that warmer conditions in the month prior to slaughter are associated with a lower incidence of cold-induced immune stress, potentially lowering the subsequent metabolic demand (Bohler et al. 2021).

### The moisture-litter-lesion axis

Arthritis (AR) and cellulitis (CE) were significantly predicted by lagged precipitation (β = 0.002) and mean humidity (β = − 0.038 to − 0.027; Table S4). In humid subtropical agro-ecologies, heavy rainfall surges are hypothesized to lead to “moist litter” or caking, which increases ammonia levels and facilitates the entry of pathogens like *Mycoplasma* and *E. coli* into the joints and subcutaneous tissues. The delay of 1–3 months between high moisture events and carcass rejection is consistent with the hypothesis that environmental conditions affecting litter quality early in the production cycle may be associated with final carcass quality. It is important to clarify that, since the broiler production cycle lasts approximately 42 days, lag effects of 2 to 3 months do not represent the same flock. Instead, they likely reflect environmental carry-over effects at the farm level. Sustained precipitation or humidity months prior may lead to cumulative deterioration of litter quality and increased pathogen pressure in the facility, which subsequently impacts the health of future flocks placed in the same environment.

Similarly, skin lesions (SL) were negatively correlated with temperature range (ρ = − 0.66) and positively with humidity (ρ = 0.31–0.38; Fig. 4). These results align with findings from high-rainfall regions in Southeast Asia, where high stocking densities combined with sustained humidity are reported to exacerbate contact dermatitis (Kang et al. 2020).

### Metabolic stress and thermoregulation

Ascites (AS) showed a high coefficient of determination (adjusted R^2^ = 0.975, Table S5), which may reflect the strong seasonality of this condition (Wideman et al. 2013), although the potential for overfitting due to the sample size (*n* = 34) must be considered. However, this high explained variance is consistent with the dominant role of thermal stress in ascites etiology. Furthermore, the robustness of this association was supported by the Durbin-Watson test (DW = 2.04), confirming residual independence, and by Ridge Regression, which yielded consistent coefficients, suggesting that the result is not a statistical artifact of multicollinearity or sample size. In the Southern Hemisphere winter, low temperatures are associated with an increased metabolic demand for oxygen to maintain thermogenesis (Kumar et al. 2021; Moreira et al. 2024). In rapidly growing broilers, this metabolic strain is a known precursor to pulmonary arterial hypertension and abdominal fluid accumulation, a pattern also documented in temperate-subtropical transition zones worldwide (Wideman et al. 2013; Belintani et al. 2019).

Airsacculitis (AI) peaked at 1.4% in 2023, showing a positive correlation with concurrent temperature (Fig. 4). This suggests a potential association where warmer conditions could exacerbate the effects of respiratory pathogens, possibly through increased dust levels or poor air quality during periods of low ventilation aimed at heat conservation.

### Economic and sustainability implications

With an estimated annual financial loss of US$ 150,000 per processing facility (Azizpour and Amirajam 2024), carcass rejections represent a significant “leakage” in the protein value chain (Salines et al. 2017). Reducing these losses directly contributes to the United Nations Sustainable Development Goals, specifically Zero Hunger (SDG 2) and Responsible Consumption and Production (SDG 12), by decreasing food waste and improving the environmental footprint per kilogram of meat produced (McManus et al. 2023).

### Limitations and future directions

Reliance on meteorological data from a single station may limit generalizability to mountainous microclimates within the region. Additionally, the model did not include farm-level variables such as stocking density, vaccination protocols, or specific genetic lineages, like Ross 308 or Hubbard, which are known to mediate environmental sensitivity. Furthermore, the ecological nature of this study, based on monthly aggregate data (*n* = 34), limits the ability to establish direct causality, reinforcing that identified patterns should be interpreted as statistical associations. The presence of temporal autocorrelation and the potential for an ‘ecological fallacy’, where population-level associations may not reflect individual farm dynamics, are inherent limitations of this modeling framework.

Future research should expand the geographic scope by incorporating grid-based climatic data and utilize machine learning algorithms, such as random forest or ARIMA models, to improve predictive accuracy for seasonal peaks. Including One Health indicators, such as the phylogenetic profiling of *E. coli* in condemned carcasses, could further link environmental management with public health safety.

## Conclusion

This study suggests that broiler carcass condemnation patterns in humid subtropical regions are influenced by both concurrent climatic conditions and environmental factors from earlier production phases. Specifically, cold stress was associated with increased ascites rates (*p* < 0.01), while lagged precipitation and humidity emerged as potential drivers for inflammatory and joint lesions (*p* ≤ 0.05).

Given the ecological and regional design of this research, it is important to emphasize that these findings represent statistical associations rather than direct evidence of causation. The absence of farm-level variables and the reliance on monthly aggregated data are inherent limitations that necessitate caution in extrapolating these results. Nevertheless, these results provide preliminary actionable insights for anticipating seasonal risks and optimizing management in humid subtropical poultry systems. Future longitudinal studies incorporating farm-specific covariates are required to validate these predictive trends and further refine environmental mitigation strategies in the poultry value chain.

## Supplementary Information

Below is the link to the electronic supplementary material.

Supplementary Material 1

Supplementary Material 2: Fig. S1: Correlation heatmap between poultry condemnation causes and climatic variables with lag of 1 month. Each cell shows the Spearman rank correlation coefficient with the corresponding p-value. P-values < 0.05 indicate statistically significant correlation.

Supplementary Material 3: Fig. S2: Correlation heatmap between poultry condemnation causes and climatic variables with lag of 2 months. Each cell shows the Spearman rank correlation coefficient with the corresponding p-value. P-values < 0.05 indicate statistically significant correlation

Supplementary Material 4: Fig. S3: Correlation heatmap between poultry condemnation causes and climatic variables with lag of 3 months. Each cell shows the Spearman rank correlation coefficient with the corresponding p-value. P-values < 0.05 indicate statistically significant correlation.

## Data Availability

The data that support the findings of this study are available from the corresponding author, WSR, upon reasonable request.
