# Supplementary material for: Environmental drivers of broiler carcass condemnation in humid subtropical regions: an exploratory study on the association of lagged climatic effects
Source: Trop Anim Health Prod. 2026 May 20;58(5):279. doi: 10.1007/s11250-026-05081-y (PMC13190353; doi:10.1007/s11250-026-05081-y)
Supplement: Supplementary file 1 — Supplementary Material 1 [file 11250_2026_5081_MOESM1_ESM.docx]

**SUPPLEMENTARY FILE**


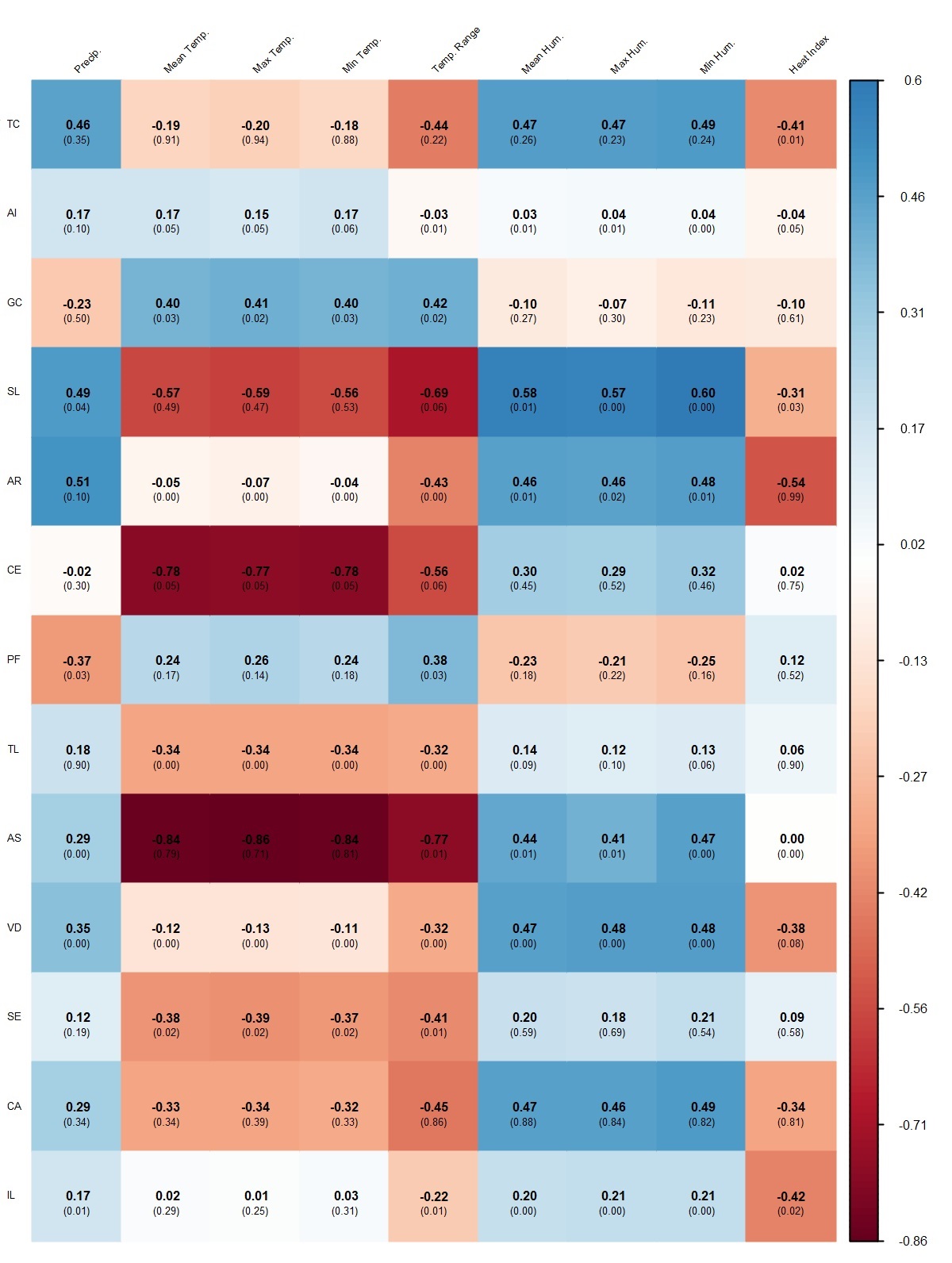


**Figure S1**

**
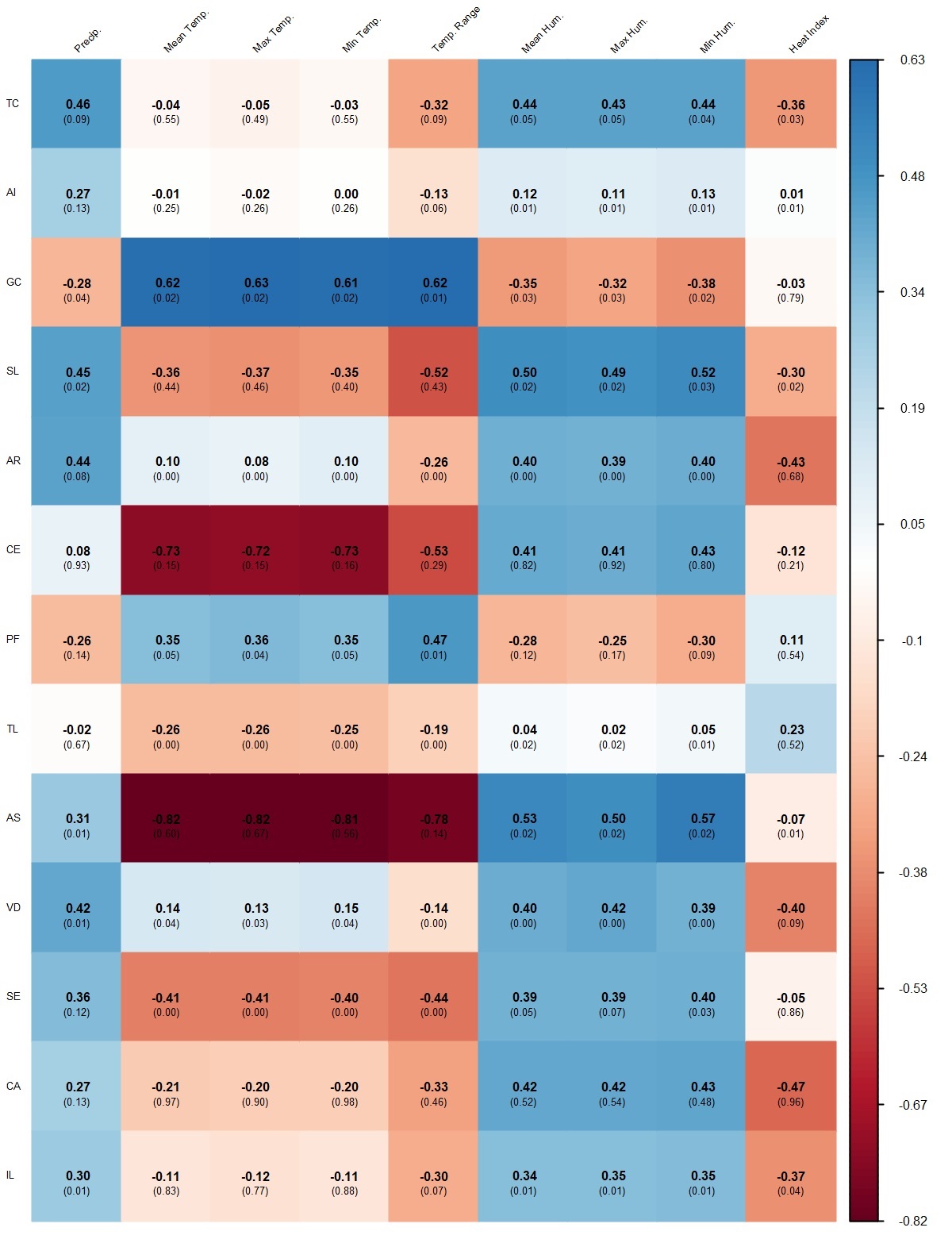
**

**Figure S2**

**
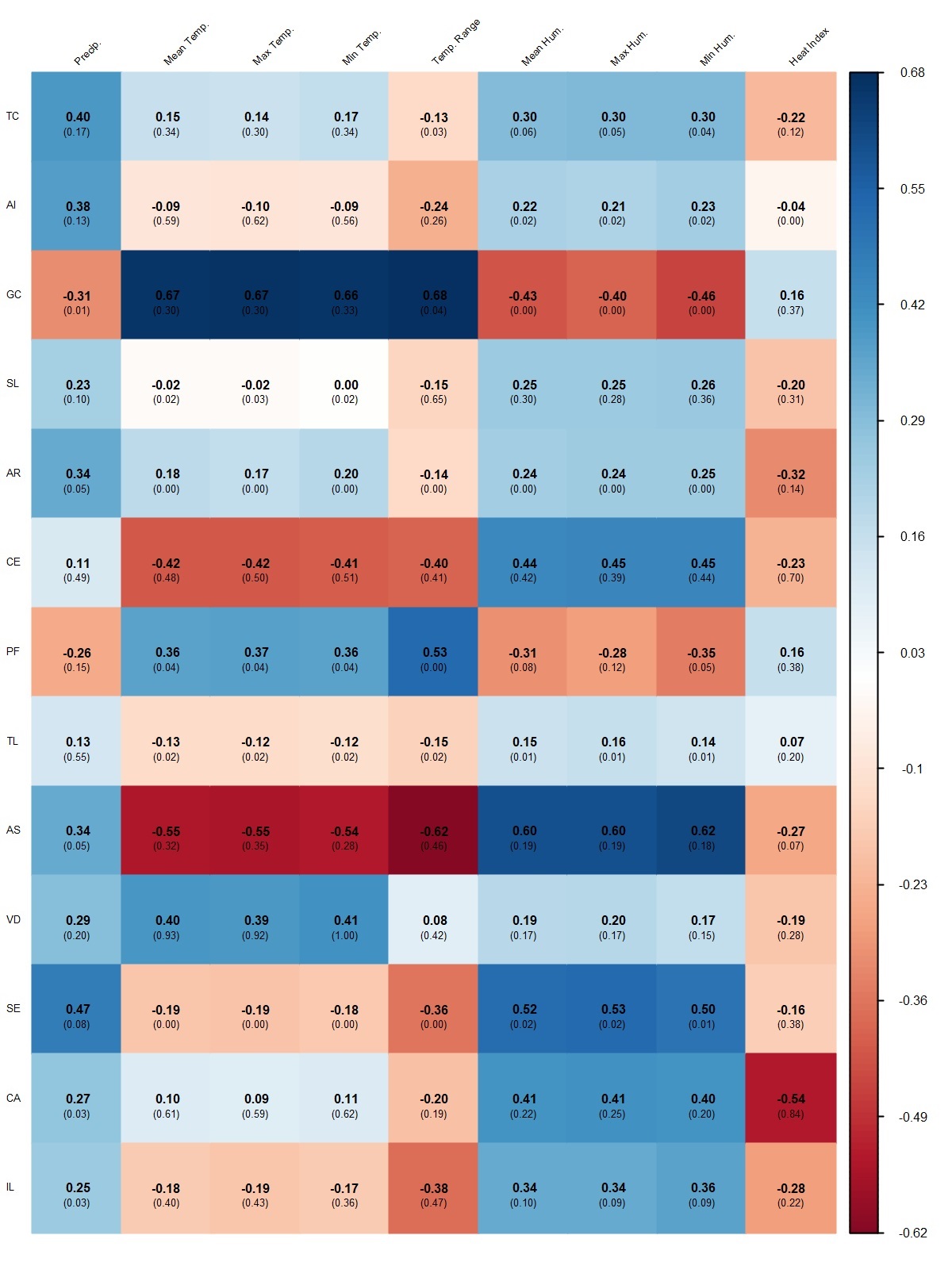
**

**Figure S3**

**Table S1. Descriptive statistics for each condemnation cause of broiler carcases in Santa Catarina from 2021 to 2023.**

| **Variable** | **Year** | **Mean** | **Standard Deviation** | **Minimum** | **Maximum** |
| --- | --- | --- | --- | --- | --- |
| Airsacculitis Proportion | 2021 | 0.0022 | 0.0007 | 0.0016 | 0.0033 |
|  | 2022 | 0.0098 | 0.0088 | 0.0025 | 0.0295 |
|  | 2023 | 0.0143 | 0.0087 | 0.0060 | 0.0295 |
| Cellulitis Proportion | 2021 | 0.0074 | 0.0015 | 0.0056 | 0.0107 |
|  | 2022 | 0.0080 | 0.0028 | 0.0050 | 0.0137 |
|  | 2023 | 0.0067 | 0.0015 | 0.0044 | 0.0094 |
| Processing Failures Proportion | 2021 | 0.0085 | 0.0021 | 0.0051 | 0.0111 |
|  | 2022 | 0.0081 | 0.0017 | 0.0062 | 0.0111 |
|  | 2023 | 0.0069 | 0.0013 | 0.0051 | 0.0089 |
| Traumatic Lesions Proportion | 2021 | 0.0034 | 0.0005 | 0.0027 | 0.0041 |
|  | 2022 | 0.0037 | 0.0006 | 0.0029 | 0.0040 |
|  | 2023 | 0.0029 | 0.0004 | 0.0024 | 0.0036 |
| Ascites Proportion | 2021 | 0.0026 | 0.0011 | 0.0018 | 0.0046 |
|  | 2022 | 0.0038 | 0.0016 | 0.0022 | 0.0060 |
|  | 2023 | 0.0034 | 0.0011 | 0.0022 | 0.0047 |
| Visual Defects Proportion | 2021 | 0.0022 | 0.0002 | 0.0019 | 0.0025 |
|  | 2022 | 0.0024 | 0.0004 | 0.0020 | 0.0029 |
|  | 2023 | 0.0026 | 0.0006 | 0.0020 | 0.0037 |
| Septicaemia Proportion | 2021 | 0.0016 | 0.0002 | 0.0012 | 0.0018 |
|  | 2022 | 0.0023 | 0.0008 | 0.0014 | 0.0033 |
|  | 2023 | 0.0016 | 0.0003 | 0.0012 | 0.0020 |
| Cachexia Proportion | 2021 | 0.0004 | 0.0001 | 0.0003 | 0.0006 |
|  | 2022 | 0.0004 | 0.0001 | 0.0003 | 0.0007 |
|  | 2023 | 0.0005 | 0.0002 | 0.0003 | 0.0008 |
| Inflammatory Lesions Proportion | 2021 | 0.0004 | 0.0002 | 0.0003 | 0.0007 |
|  | 2022 | 0.0006 | 0.0003 | 0.0002 | 0.0010 |
|  | 2023 | 0.0007 | 0.0003 | 0.0005 | 0.0014 |

**Table S2. Descriptive statistics of the meteorological variables in Santa Catarina from 2021 to 2023.**

| **Variable** | **Year** | **Mean** | **Standard Deviation** | **Minimum** | **Maximum** |
| --- | --- | --- | --- | --- | --- |
| Precipitation (mm) | 2021 | 99.30 | 94.33 | 7.40 | 323.60 |
|  | 2022 | 178.76 | 92.66 | 40.78 | 370.00 |
|  | 2023 | 215.49 | 142.13 | 88.40 | 545.80 |
| Mean Temperature (°C) | 2021 | 19.93 | 3.72 | 13.43 | 24.60 |
|  | 2022 | 18.12 | 3.86 | 13.70 | 24.17 |
|  | 2023 | 20.00 | 3.10 | 15.55 | 23.83 |
| Maximum Temperature (°C) | 2021 | 20.58 | 3.86 | 14.07 | 25.44 |
|  | 2022 | 18.68 | 3.98 | 14.18 | 24.93 |
|  | 2023 | 20.62 | 3.21 | 16.01 | 24.54 |
| Minimum Temperature (°C) | 2021 | 19.28 | 3.59 | 12.79 | 23.81 |
|  | 2022 | 17.58 | 3.75 | 13.21 | 23.44 |
|  | 2023 | 19.39 | 3.00 | 15.07 | 23.12 |
| Mean Temperature Range (°C) | 2021 | 1.30 | 0.30 | 0.88 | 1.63 |
|  | 2022 | 1.10 | 0.25 | 0.67 | 1.49 |
|  | 2023 | 1.23 | 0.36 | 0.87 | 1.52 |
| Mean Relative Humidity (%) | 2021 | 66.94 | 5.95 | 55.66 | 73.06 |
|  | 2022 | 71.85 | 7.91 | 58.43 | 82.81 |
|  | 2023 | 73.66 | 3.49 | 69.50 | 79.05 |
| Maximum Relative Humidity (%) | 2021 | 70.14 | 5.90 | 58.99 | 76.27 |
|  | 2022 | 74.95 | 7.77 | 61.62 | 85.13 |
|  | 2023 | 76.69 | 3.49 | 72.42 | 81.45 |
| Minimum Relative Humidity (%) | 2021 | 63.77 | 6.03 | 52.27 | 69.89 |
|  | 2022 | 68.78 | 8.04 | 55.47 | 80.46 |
|  | 2023 | 70.73 | 3.48 | 66.63 | 76.68 |
| Heat Index | 2021 | 76.76 | 4.36 | 73.06 | 86.14 |
|  | 2022 | 74.73 | 2.74 | 71.17 | 78.59 |
|  | 2023 | 74.27 | 2.00 | 70.39 | 76.80 |

**Table S3. Detailed regression results for total condemnations rate (TC), airsacculitis rate (AI), gastrointestinal contamination rate (GC), and skin lesions rate (SL)**

|  | TC | | AI | | GC | | SL | | |
| --- | --- | --- | --- | --- | --- | --- | --- | --- | --- |
|  | Base Model | Stepwise Model | Base Model | Stepwise Model | Base Model | Stepwise Model | Base Model | Stepwise Model | |
| β_0_ | 50.628 (23.481) | 50.522 (17.771) | 27.015 (12.590)^*^ | 27.015 (12.590)^*^ | -13.435 (11.206) | -7.497 (6.916) | 4.404 (16.485) | 5.504 (0.338)^***^ | |
| β_1_ | -0.004 (0.003) | -0.004 (0.003) | -0.002 (0.002) | -0.002 (0.002) | -0.0002 (0.001) |  | -0.001 (0.002) |  | |
| β_2_ | -0.141 (0.155) | -0.141 (0.146) | -0.250 (0.083)^**^ | -0.250 (0.083)^**^ | 0.119 (0.074) | 0.078 (0.043)^*^ | 0.023 (0.109) |  | |
| β_3_ | -0.100 (0.070) | -0.100 (0.063) | -0.071 (0.038)^*^ | -0.071 (0.038)^*^ | 0.020 (0.033) |  | 0.009 (0.049) |  | |
| β_4_ | -0.094 (0.099) | -0.094 (0.094) | -0.050 (0.053) | -0.050 (0.053) | 0.031 (0.047) |  | -0.026 (0.070) |  | |
| β_5_ | 0.00003 (0.004) |  | -0.002 (0.002) | -0.002 (0.002) | -0.002 (0.002) | -0.002 (0.001) | 0.001 (0.003) |  | |
| β_6_ | -0.259 (0.176) | -0.258 (0.148) | -0.192 (0.094)^*^ | -0.192 (0.094)^*^ | 0.141 (0.084) | 0.132 (0.072)^*^ | -0.038 (0.123) | -0.121 (0.017)^***^ | |
| β_7_ | -0.076 (0.089) | -0.075 (0.049) | -0.055 (0.048) | -0.055 (0.048) | 0.052 (0.042) | 0.042 (0.034) | 0.001 (0.062) |  | |
| β_8_ | -0.124 (0.111) | -0.123 (0.093) | -0.066 (0.059) | -0.066 (0.059) | 0.086 (0.053) | 0.078 (0.046) | 0.002 (0.078) |  | |
| Jan. | -1.564 (1.277) | -1.561 (1.189) | 0.706 (0.685) | 0.706 (0.685) | -0.825 (0.609) | -0.631 (0.401) | -0.197 (0.896) |  | |
| Feb. | -1.546 (1.035) | -1.544 (0.976) | 0.569 (0.555) | 0.569 (0.555) | -1.184 (0.494)^**^ | -1.054 (0.356)^***^ | -0.226 (0.727) |  | |
| Mar. | -0.092 (0.853) | -0.092 (0.819) | 0.909 (0.458)^*^ | 0.909 (0.458)^*^ | -0.788 (0.407)^*^ | -0.613 (0.278)^**^ | -0.284 (0.599) |  | |
| Apr. |  |  |  |  |  |  |  |  | |
| May | -0.680 (1.028) | -0.676 (0.858) | -1.300 (0.551)^**^ | -1.300 (0.551)^**^ | 0.775 (0.491) | 0.693 (0.418) | 0.069 (0.722) |  | |
| Jun. | -0.152 (1.205) | -0.148 (1.047) | -1.472 (0.646)^**^ | -1.472 (0.646)^**^ | 0.967 (0.575) | 0.928 (0.514)^*^ | 0.452 (0.846) |  | |
| Jul. | -1.526 (1.223) | -1.522 (1.083) | -1.975 (0.656)^**^ | -1.975 (0.656)^**^ | 0.362 (0.584) | 0.315 (0.477) | 0.806 (0.859) |  | |
| Aug. | -2.184 (1.287) | -2.179 (1.059)^*^ | -2.097 (0.690)^**^ | -2.097 (0.690)^**^ | -0.166 (0.614) | -0.263 (0.484) | 0.839 (0.904) |  | |
| Sep. | -2.642 (1.240)^*^ | -2.637 (1.008)^**^ | -1.429 (0.665)^*^ | -1.429 (0.665)^*^ | -0.282 (0.592) | -0.327 (0.489) | 0.329 (0.870) |  | |
| Oct. | -0.749 (1.155) | -0.747 (1.059) | 0.006 (0.619) | 0.006 (0.619) | -0.297 (0.551) | -0.369 (0.382) | 0.320 (0.811) |  | |
| Nov. | -1.270 (1.715) | -1.260 (0.950) | 0.699 (0.920) | 0.699 (0.920) | 0.291 (0.819) | 0.111 (0.598) | -0.082 (1.204) |  | |
| Dec. | -1.161 (1.399) | -1.154 (1.020) | 0.710 (0.750) | 0.710 (0.750) | -0.434 (0.668) | -0.390 (0.481) | -0.043 (0.982) |  | |
| 2022 | 2.422 (0.399)^***^ | 2.423 (0.378)^***^ | 1.114 (0.214)^***^ | 1.114 (0.214)^***^ | 0.011 (0.190) | -0.021 (0.152) | 0.453 (0.280) | 0.462 (0.142)^***^ | |
| 2023 | 4.325 (0.454)^***^ | 4.325 (0.435)^***^ | 1.956 (0.243)^***^ | 1.956 (0.243)^***^ | -0.192 (0.217) | -0.202 (0.169) | 0.862 (0.318)^**^ | 1.018 (0.143)^***^ | |
| Adj. R^2^ | 0.829 | 0.843 | 0.739 | 0.739 | 0.521 | 0.599 | 0.490 | 0.721 | |
| RSE (df) | 0.636 (12) | 0.611 (13) | 0.341 (12) | 0.341 (12) | 0.304 (12) | 0.278 (15) | 0.447 (12) | 0.330 (30) | |
| SW (p) | 0.8491 | | 0.6314 | | 0.2043 | | 0.9843 | | |
| BP (p) | 0.3547 | | 0.1881 | | 0.2394 | | 0.1772 | | |
| DW | 1.88 (p = 0.322) | | 1.95 (p = 0.410) | | 1.82 (p = 0.285) | | 1.84 (p = 0.291) | | |
| Ridge Consist. | Confirmed | | Confirmed | | Confirmed | | Confirmed | | |
|  | GVIF (Stepwise) | GVIF^1/(2df)^ (Stepwise) | GVIF (Stepwise) | GVIF^1/(2df)^ (Stepwise) | GVIF (Stepwise) | GVIF^1/(2df)^ (Stepwise) | GVIF (Stepwise) | | GVIF^1/(2df)^ (Stepwise) |
| X_1_ | 10.30 | 3.21 | 10.50 | 3.24 |  |  |  | |  |
| X_2_ | 22.92 | 4.79 | 23.84 | 4.88 | 9.53 | 3.09 |  | |  |
| X_3_ | 16.16 | 4.02 | 18.61 | 4.31 |  |  |  | |  |
| X_4_ | 6.45 | 2.54 | 6.61 | 2.57 |  |  |  | |  |
| X_5_ |  |  | 18.13 | 4.26 | 10.68 | 3.27 |  | |  |
| X_6_ | 22.67 | 4.76 | 29.52 | 5.43 | 25.91 | 5.09 | 1.03 | | 1.01 |
| X_7_ | 9.97 | 3.16 | 29.89 | 5.47 | 22.73 | 4.77 |  | |  |
| X_8_ | 6.26 | 2.50 | 8.23 | 2.87 | 7.52 | 2.74 |  | |  |
| Month | 5798.29 | 1.48 | 30520.37 | 1.60 | 908.70 | 1.36 |  | |  |
| Year | 4.15 | 1.43 | 4.28 | 1.44 | 2.84 | 1.30 | 1.03 | | 1.01 |

RSE: Residual standard error; SW: Shapiro Wilk; BP: Breusch-Pagan; df: degrees of freedom; ^*^p < 0.1; ^**^p < 0.05; ^***^p < 0.01; GVIF: Generalised variance inflation factor. Regression coefficients are expressed as mean (standard error). X_1_: precipitation (mm), X_2_: mean temperature (°C), X_3_: mean humidity (%), X_4_: heat index, X_5_: precipitation lag-1 (mm), X_6_: temperature lag-1 (°C), X_7_: humidity lag-1 (%), X_8_: heat index lag-1. Note: Model robustness was verified using the Durbin-Watson (DW) test for residual independence and Ridge Regression Consistency for coefficient stability under multicollinearity (VIF > 5). Sample size n = 34 months

**Table S4. Detailed regression results for total arthritis rate (AR), cellulitis rate (CE), processing failure rate (PF), and traumatic lesions rate (TL)**

|  | AR | | CE | | PF | | TL | | |
| --- | --- | --- | --- | --- | --- | --- | --- | --- | --- |
|  | Base Model | Stepwise Model | Base Model | Stepwise Model | Base Model | Stepwise Model | Base Model | Stepwise Model | |
| β_0_ | 13.353 (10.106) | 17.228 (7.022)^**^ | 7.447 (4.392) | 8.116 (2.804)^**^ | 7.163 (4.660) | 6.229 (1.429)^***^ | -0.374 (0.921) | -0.059 (0.096) | |
| β_1_ | -0.001 (0.001) |  | -0.0003 (0.001) | -0.0005 (0.0004) | 0.0001 (0.001) |  | 0.00004 (0.0001) |  | |
| β_2_ | 0.029 (0.067) |  | 0.021 (0.029) |  | -0.006 (0.031) |  | 0.008 (0.006) | 0.011 (0.003)^***^ | |
| β_3_ | -0.021 (0.030) | -0.038 (0.015)^**^ | -0.001 (0.013) |  | -0.011 (0.014) | -0.010 (0.006) | -0.001 (0.003) |  | |
| β_4_ | 0.021 (0.043) |  | 0.009 (0.019) |  | -0.041 (0.020)^*^ | -0.038 (0.012)^***^ | -0.002 (0.004) |  | |
| β_5_ | 0.002 (0.002) | 0.002 (0.001)^**^ | 0.002 (0.001)^**^ | 0.002 (0.001)^**^ | 0.0004 (0.001) | 0.0004 (0.0003) | 0.0002 (0.0002) | 0.0002 (0.0001)^***^ | |
| β_6_ | -0.098 (0.076) | -0.107 (0.067) | -0.059 (0.033)^*^ | -0.053 (0.029)^*^ | -0.008 (0.035) |  | 0.011 (0.007) | 0.006 (0.003)^*^ | |
| β_7_ | -0.055 (0.038) | -0.057 (0.034) | -0.029 (0.017) | -0.027 (0.014)^**^ | -0.002 (0.018) |  | 0.002 (0.003) |  | |
| β_8_ | -0.089 (0.048)^*^ | -0.093 (0.043)^**^ | -0.024 (0.021) | -0.023 (0.019) | -0.025 (0.022) | -0.022 (0.011)^*^ | 0.004 (0.004) |  | |
| Jan. | -1.656 (0.550)^**^ | -1.479 (0.370)^***^ | -0.400 (0.239) | -0.244 (0.150) | 0.248 (0.253) | 0.214 (0.134) | -0.015 (0.050) | -0.039 (0.027) | |
| Feb. | -0.877 (0.446)^*^ | -0.724 (0.323)^**^ | -0.351 (0.194)^*^ | -0.240 (0.138) | 0.166 (0.205) | 0.128 (0.126) | -0.058 (0.041) | -0.065 (0.025)^**^ | |
| Mar. | -0.283 (0.367) | -0.122 (0.226) | -0.301 (0.160)^*^ | -0.233 (0.103)^**^ | 0.227 (0.169) | 0.202 (0.094)^**^ | -0.033 (0.033) | -0.049 (0.021)^**^ | |
| Apr. |  |  |  |  |  |  |  |  | |
| May | 0.104 (0.443) | 0.044 (0.365) | -0.294 (0.192) | -0.318 (0.160)^*^ | -0.038 (0.204) | 0.001 (0.096) | 0.086 (0.040)^*^ | 0.062 (0.023)^**^ | |
| Jun. | 0.170 (0.519) | 0.145 (0.434) | -0.332 (0.225) | -0.375 (0.193)^*^ | -0.052 (0.239) | -0.002 (0.111) | 0.129 (0.047)^**^ | 0.100 (0.030)^***^ | |
| Jul. | -0.553 (0.526) | -0.593 (0.439) | -0.388 (0.229) | -0.371 (0.187)^*^ | -0.053 (0.243) | 0.00000 (0.114) | 0.131 (0.048)^**^ | 0.107 (0.032)^***^ | |
| Aug. | -0.692 (0.554) | -0.751 (0.465) | -0.486 (0.241)^*^ | -0.464 (0.192)^**^ | -0.057 (0.256) | -0.009 (0.115) | 0.117 (0.050)^**^ | 0.093 (0.028)^***^ | |
| Sep. | -0.981 (0.534)^*^ | -1.008 (0.472)^**^ | -0.521 (0.232)^**^ | -0.467 (0.197)^**^ | -0.148 (0.246) | -0.110 (0.100) | 0.093 (0.049)^*^ | 0.063 (0.025)^**^ | |
| Oct. | -0.327 (0.497) | -0.529 (0.348) | -0.349 (0.216) | -0.307 (0.172)^*^ | -0.294 (0.229) | -0.256 (0.093)^**^ | 0.045 (0.045) | 0.033 (0.021) | |
| Nov. | -1.271 (0.738) | -1.336 (0.665)^*^ | -0.992 (0.321)^***^ | -0.866 (0.246)^***^ | -0.461 (0.340) | -0.431 (0.148)^**^ | -0.024 (0.067) | -0.064 (0.029)^**^ | |
| Dec. | -1.145 (0.602)^*^ | -1.036 (0.503)^*^ | -0.856 (0.262)^***^ | -0.696 (0.192)^***^ | -0.146 (0.278) | -0.153 (0.116) | -0.035 (0.055) | -0.073 (0.026)^**^ | |
| 2022 | 1.007 (0.172)^***^ | 0.957 (0.141)^***^ | -0.241 (0.075)^***^ | -0.263 (0.063)^***^ | -0.232 (0.079)^**^ | -0.222 (0.062)^***^ | 0.049 (0.016)^***^ | 0.048 (0.011)^***^ | |
| 2023 | 2.151 (0.195)^***^ | 2.106 (0.158)^***^ | -0.327 (0.085)^***^ | -0.333 (0.072)^***^ | -0.476 (0.090)^***^ | -0.475 (0.077)^***^ | -0.045 (0.018)^**^ | -0.044 (0.013)^***^ | |
| Adj. R^2^ | 0.901 | 0.917 | 0.580 | 0.633 | 0.610 | 0.705 | 0.646 | 0.718 | |
| RSE (df) | 0.274 (12) | 0.250 (15) | 0.119 (12) | 0.111 (15) | 0.126 (12) | 0.110 (16) | 0.025 (12) | 0.022 (17) | |
| SW (p) | 0.5887 | | 0.7631 | | 0.8389 | | 0.9295 | | |
| BP (p) | 0.1797 | | 0.4639 | | 0.2937 | | 0.0120 | | |
| DW | 1.72 (p = 0.185) | | 1.91 (p = 0.380) | | 1.78 (p = 0.215) | | 1.92 (p = 0.395) | | |
| Ridge Consist. | Confirmed | | Confirmed | | Confirmed | | Confirmed | | |
|  | GVIF (Stepwise) | GVIF^1/(2df)^ (Stepwise) | GVIF (Stepwise) | GVIF^1/(2df)^ (Stepwise) | GVIF (Stepwise) | GVIF^1/(2df)^ (Stepwise) | GVIF (Stepwise) | | GVIF^1/(2df)^ (Stepwise) |
| X_1_ |  |  | 5.65 | 2.38 |  |  |  | |  |
| X_2_ |  |  |  |  |  |  | 9.52 | | 3.09 |
| X_3_ | 5.70 | 2.39 |  |  | 5.17 | 2.27 |  | |  |
| X_4_ |  |  |  |  | 3.30 | 1.82 |  | |  |
| X_5_ | 16.64 | 4.08 | 15.63 | 3.95 | 4.90 | 2.21 | 3.82 | | 1.95 |
| X_6_ | 27.93 | 5.29 | 25.85 | 5.08 |  |  | 8.35 | | 2.89 |
| X_7_ | 28.33 | 5.32 | 25.52 | 5.05 |  |  |  | |  |
| X_8_ | 8.05 | 2.84 | 7.65 | 2.77 | 2.72 | 1.65 |  | |  |
| Month | 1188.62 | 1.38 | 1137.69 | 1.38 | 42.21 | 1.19 | 105.35 | | 1.24 |
| Year | 2.75 | 1.29 | 2.84 | 1.30 | 2.78 | 1.29 | 2.38 | | 1.24 |

RSE: Residual standard error; SW: Shapiro Wilk; BP: Breusch-Pagan; df: degrees of freedom; ^*^p < 0.1; ^**^p < 0.05; ^***^p < 0.01; GVIF: Generalised variance inflation factor. Regression coefficients are expressed as mean (standard error). X_1_: precipitation (mm), X_2_: mean temperature (°C), X_3_: mean humidity (%), X_4_: heat index, X_5_: precipitation lag-1 (mm), X_6_: temperature lag-1 (°C), X_7_: humidity lag-1 (%), X_8_: heat index lag-1. Note: Model robustness was verified using the Durbin-Watson (DW) test for residual independence and Ridge Regression Consistency for coefficient stability under multicollinearity (VIF > 5). Sample size n = 34 months

**Table S5. Detailed regression results for total ascites rate (AS), visual defects rate (VD), septicaemia rate (SE), and cachexia rate (CA)**

|  | AS | | VD | | SE | | CA | | |
| --- | --- | --- | --- | --- | --- | --- | --- | --- | --- |
|  | Base Model | Stepwise Model | Base Model | Stepwise Model | Base Model | Stepwise Model | Base Model | Stepwise Model | |
| β_0_ | 1.795 (0.584)^***^ | 1.962 (0.252)^***^ | 1.574 (0.602)^**^ | 1.574 (0.602)^**^ | 1.133 (1.172) | 0.634 (0.161)^***^ | 0.303 (0.243) | 0.186 (0.043)^***^ | |
| β_1_ | -0.00004 (0.0001) |  | -0.0001 (0.0001) | -0.0001 (0.0001) | -0.00002 (0.0001) |  | 0.00004 (0.00003) | 0.00003 (0.00002) | |
| β_2_ | -0.020 (0.004)^***^ | -0.021 (0.003)^***^ | -0.005 (0.004) | -0.005 (0.004) | -0.021 (0.008)^**^ | -0.018 (0.005)^***^ | -0.003 (0.002) | -0.003 (0.001)^***^ | |
| β_3_ | -0.006 (0.002)^***^ | -0.006 (0.001)^***^ | -0.003 (0.002) | -0.003 (0.002) | -0.003 (0.004) | -0.002 (0.002) | -0.002 (0.001)^*^ | -0.001 (0.0005)^**^ | |
| β_4_ | -0.008 (0.002)^***^ | -0.008 (0.002)^***^ | -0.004 (0.003) | -0.004 (0.003) | -0.004 (0.005) |  | 0.00001 (0.001) |  | |
| β_5_ | 0.00005 (0.0001) |  | 0.0001 (0.0001) | 0.0001 (0.0001) | -0.0001 (0.0002) | -0.0001 (0.0001) | -0.00002 (0.00004) | -0.00003 (0.00002) | |
| β_6_ | -0.015 (0.004)^***^ | -0.017 (0.002)^***^ | -0.008 (0.005) | -0.008 (0.005) | -0.001 (0.009) |  | -0.001 (0.002) |  | |
| β_7_ | 0.0002 (0.002) |  | -0.004 (0.002) | -0.004 (0.002) | 0.0001 (0.004) |  | -0.0005 (0.001) |  | |
| β_8_ | 0.001 (0.003) |  | -0.004 (0.003) | -0.004 (0.003) | -0.002 (0.006) |  | -0.001 (0.001) |  | |
| Jan. | 0.117 (0.032)^***^ | 0.118 (0.025)^***^ | -0.026 (0.033) | -0.026 (0.033) | 0.106 (0.064) | 0.065 (0.033)^*^ | -0.008 (0.013) | -0.006 (0.007) | |
| Feb. | 0.071 (0.026)^**^ | 0.080 (0.020)^***^ | -0.038 (0.027) | -0.038 (0.027) | 0.060 (0.052) | 0.029 (0.031) | -0.013 (0.011) | -0.014 (0.007)^**^ | |
| Mar. | 0.059 (0.021)^**^ | 0.061 (0.017)^***^ | -0.010 (0.022) | -0.010 (0.022) | 0.057 (0.043) | 0.040 (0.027) | 0.0004 (0.009) | 0.001 (0.006) | |
| Apr. |  |  |  |  |  |  |  |  | |
| May | -0.028 (0.026) | -0.036 (0.015)^**^ | -0.032 (0.026) | -0.032 (0.026) | -0.048 (0.051) | -0.043 (0.026) | -0.004 (0.011) | 0.002 (0.006) | |
| Jun. | -0.014 (0.030) | -0.022 (0.030) | -0.016 (0.031) | -0.016 (0.031) | -0.060 (0.060) | -0.054 (0.031)^*^ | 0.007 (0.012) | 0.015 (0.007)^**^ | |
| Jul. | 0.018 (0.030) | 0.010 (0.022) | -0.042 (0.031) | -0.042 (0.031) | -0.038 (0.061) | -0.036 (0.029) | -0.004 (0.013) | 0.005 (0.006) | |
| Aug. | 0.066 (0.032)^*^ | 0.058 (0.019)^***^ | -0.091 (0.033)^**^ | -0.091 (0.033)^**^ | 0.007 (0.064) | 0.005 (0.027) | -0.019 (0.013) | -0.010 (0.006) | |
| Sep. | 0.091 (0.031)^**^ | 0.082 (0.017)^***^ | -0.095 (0.032)^**^ | -0.095 (0.032)^**^ | 0.045 (0.062) | 0.041 (0.023)^*^ | -0.020 (0.013) | -0.012 (0.005)^**^ | |
| Oct. | 0.107 (0.029)^***^ | 0.091 (0.014)^***^ | -0.051 (0.030) | -0.051 (0.030) | 0.018 (0.058) | 0.016 (0.023) | -0.014 (0.012) | -0.007 (0.007) | |
| Nov. | 0.056 (0.043) | 0.058 (0.017)^***^ | -0.077 (0.044) | -0.077 (0.044) | 0.071 (0.086) | 0.066 (0.038) | -0.009 (0.018) | 0.0003 (0.008) | |
| Dec. | 0.086 (0.035)^**^ | 0.089 (0.022)^***^ | -0.037 (0.036) | -0.037 (0.036) | 0.126 (0.070)^*^ | 0.099 (0.032)^***^ | 0.00004 (0.014) | 0.006 (0.007) | |
| 2022 | 0.066 (0.010)^***^ | 0.065 (0.008)^***^ | 0.049 (0.010)^***^ | 0.049 (0.010)^***^ | 0.078 (0.020)^***^ | 0.085 (0.015)^***^ | 0.005 (0.004) | 0.005 (0.003) | |
| 2023 | 0.088 (0.011)^***^ | 0.088 (0.009)^***^ | 0.063 (0.012)^***^ | 0.063 (0.012)^***^ | 0.044 (0.023)^*^ | 0.051 (0.018)^**^ | 0.025 (0.005)^***^ | 0.025 (0.004)^***^ | |
| Adj. R^2^ | 0.970 | 0.975 | 0.744 | 0.744 | 0.598 | 0.694 | 0.724 | 0.781 | |
| RSE (df) | 0.016 (12) | 0.014 (16) | 0.016 (12) | 0.016 (12) | 0.032 (12) | 0.028 (17) | 0.007 (12) | 0.006 (16) | |
| SW (p) | 0.4234 | | 0.5400 | | 0.3881 | | 0.8094 | | |
| BP (p) | 0.3170 | | 0.1067 | | 0.1157 | | 0.3817 | | |
| DW | 2.04 (p = 0.514) | | 1.81 (p = 0.265) | | 1.76 (p = 0.198) | | 1.89 (p = 0.335) | | |
| Ridge Consist. | Confirmed | | Confirmed | | Confirmed | | Confirmed | | |
|  | GVIF (Stepwise) | GVIF^1/(2df)^ (Stepwise) | GVIF (Stepwise) | GVIF^1/(2df)^ (Stepwise) | GVIF (Stepwise) | GVIF^1/(2df)^ (Stepwise) | GVIF (Stepwise) | | GVIF^1/(2df)^ (Stepwise) |
| X_1_ |  |  | 10.50 | 3.24 |  |  | 8.86 | | 2.98 |
| X_2_ | 20.31 | 4.51 | 23.84 | 4.88 | 10.63 | 3.26 | 11.39 | | 3.38 |
| X_3_ | 6.90 | 2.63 | 18.61 | 4.31 | 5.00 | 2.24 | 10.11 | | 3.18 |
| X_4_ | 5.83 | 2.42 | 6.61 | 2.57 |  |  |  | |  |
| X_5_ |  |  | 18.13 | 4.26 | 4.40 | 2.10 | 4.66 | | 2.16 |
| X_6_ | 8.30 | 2.88 | 29.52 | 5.43 |  |  |  | |  |
| X_7_ |  |  | 29.89 | 5.47 |  |  |  | |  |
| X_8_ |  |  | 8.23 | 2.87 |  |  |  | |  |
| Month | 199.98 | 1.27 | 30520.37 | 1.60 | 79.61 | 1.22 | 298.00 | | 1.30 |
| Year | 2.73 | 1.29 | 4.28 | 1.44 | 2.64 | 1.27 | 2.69 | | 1.28 |

RSE: Residual standard error; SW: Shapiro Wilk; BP: Breusch-Pagan; df: degrees of freedom; ^*^p < 0.1; ^**^p < 0.05; ^***^p < 0.01; GVIF: Generalised variance inflation factor. Regression coefficients are expressed as mean (standard error). X_1_: precipitation (mm), X_2_: mean temperature (°C), X_3_: mean humidity (%), X_4_: heat index, X_5_: precipitation lag-1 (mm), X_6_: temperature lag-1 (°C), X_7_: humidity lag-1 (%), X_8_: heat index lag-1. Note: Model robustness was verified using the Durbin-Watson (DW) test for residual independence and Ridge Regression Consistency for coefficient stability under multicollinearity (VIF > 5). Sample size n = 34 months

**Table S6. Detailed regression results for inflammatory lesions rate (IL)**

|  | IL | | | |
| --- | --- | --- | --- | --- |
|  | Base Model | Stepwise Model | GLM Model | GLM Model |
| β_0_ | 3.691 (2.505) | 1.035 (0.384)^**^ |  | 7.601 (4.392)^*^ |
| β_1_ | 0.0001 (0.0003) |  |  |  |
| β_2_ | -0.010 (0.017) |  |  |  |
| β_3_ | -0.005 (0.007) |  |  |  |
| β_4_ | -0.007 (0.011) | -0.005 (0.004) |  | -0.034 (0.044) |
| β_5_ | 0.0002 (0.0004) |  |  |  |
| β_6_ | -0.022 (0.019) |  |  |  |
| β_7_ | -0.008 (0.009) |  |  |  |
| β_8_ | -0.019 (0.012) | -0.007 (0.003)^**^ |  | -0.096 (0.047)^*^ |
| Jan. | 0.017 (0.136) |  |  |  |
| Feb. | 0.022 (0.110) |  |  |  |
| Mar. | 0.046 (0091) |  |  |  |
| Apr. |  |  |  |  |
| May | -0.134 (0.110) |  |  |  |
| Jun. | -0.172 (0.129) |  |  |  |
| Jul. | -0.165 (0.130) |  |  |  |
| Aug. | -0.139 (0.137) |  |  |  |
| Sep. | -0.137 (0.132) |  |  |  |
| Oct. | -0.133 (0.123) |  |  |  |
| Nov. | -0.202 (0.183) |  |  |  |
| Dec. | -0.077 (0.149) |  |  |  |
| 2022 | -0.069 (0.043) |  |  | -0.789 (0.299)^**^ |
| 2023 | -0.011 (0.048) |  |  | -0.184 (0.273) |
| Adj. R^2^ | -0.471 | 0.198 |  |  |
| RSE (df) | 0.068 | 0.050 | Dispersion Parameter ($\phi$) | 1.42 |
| SW (p) | 0.0769 | |  | |
| BP (p) | 0.0010 | |  | |
| Zero-Inflation |  | | Not Significant | |
|  | GVIF (Stepwise) | GVIF^1/(2df)^ (Stepwise) | GVIF (GLM) | GVIF^1/(2df)^ (GLM) |
| X_1_ | 1.78 | 1.33 |  |  |
| X_2_ |  |  |  |  |
| X_3_ |  |  |  |  |
| X_4_ | 1.61 | 1.27 | 1.22 | 1.11 |
| X_5_ |  |  |  |  |
| X_6_ |  |  |  |  |
| X_7_ |  |  |  |  |
| X_8_ | 1.21 | 1.10 | 1.14 | 1.07 |
| Month |  |  |  |  |
| Year | 1.31 | 1.07 | 1.22 | 1.05 |

RSE: Residual standard error; SW: Shapiro Wilk; BP: Breusch-Pagan; df: degrees of freedom; ^*^p < 0.1; ^**^p < 0.05; ^***^p < 0.01; GVIF: Generalised variance inflation factor. Regression coefficients are expressed as mean (standard error). X_1_: precipitation (mm), X_2_: mean temperature (°C), X_3_: mean humidity (%), X_4_: heat index, X_5_: precipitation lag-1 (mm), X_6_: temperature lag-1 (°C), X_7_: humidity lag-1 (%), X_8_: heat index lag-1. Note: The Inflammatory Lesions model was fitted using a Quasibinomial GLM to account for overdispersion in proportional data ($\phi$ = 1.42); A p-value of 0.051 for $X_{8}$ is reported as a marginal predictor. Formal testing for zero-inflation was performed (e.g., score test), showing no significant improvement in model fit (p > 0.05), thus justifying the standard GLM approach. Sample size n = 34 months

**Table S7. Ridge regression coefficients for CT, AI, GC, SL, AR, CE, PF, TL, and AS, addressing multicollinearity in stepwise linear models. Coefficients are rounded to three decimal places.**

| **Response** | **CT** | **AI** | **GC** | **SL** | **AR** | **CE** | **PF** | **TL** | **AS** |
| --- | --- | --- | --- | --- | --- | --- | --- | --- | --- |
| Intercept | 30.667 | 9.883 | 5.409 | 4.187 | 8.102 | 0.971 | 1.682 | 0.382 | 0.615 |
| X_1_ | -0.003 | -0.002 | 0.000 | 0.000 | 0.000 | 0.000 | 0.000 | 0.000 | 0.000 |
| X_2_ | -0.045 | -0.109 | 0.003 | -0.010 | -0.002 | -0.001 | 0.002 | 0.002 | -0.007 |
| X_3_ | -0.030 | -0.030 | 0.000 | 0.006 | -0.004 | -0.002 | -0.001 | -0.001 | -0.002 |
| X_4_ | -0.107 | -0.107 | -0.005 | -0.016 | -0.023 | 0.001 | -0.009 | -0.002 | -0.001 |
| X_5_ | -0.001 | -0.001 | 0.000 | 0.000 | 0.000 | 0.000 | 0.000 | 0.000 | 0.000 |
| X_6_ | -0.079 | -0.079 | 0.023 | -0.034 | -0.020 | -0.011 | 0.007 | 0.001 | -0.013 |
| X_7_ | 0.019 | 0.019 | -0.001 | 0.010 | 0.001 | 0.002 | 0.002 | 0.000 | 0.001 |
| X_8_ | -0.074 | -0.074 | -0.003 | 0.002 | -0.057 | 0.001 | -0.004 | 0.001 | 0.002 |
| January | -0.259 | 0.355 | 0.064 | -0.016 | -0.522 | -0.016 | 0.002 | 0.005 | 0.024 |
| February | -0.701 | 0.119 | -0.108 | -0.050 | -0.134 | -0.038 | -0.003 | -0.021 | 0.000 |
| March | -0.067 | 0.466 | -0.017 | -0.192 | 0.031 | 0.001 | 0.133 | -0.022 | -0.015 |
| April |  |  |  |  |  |  |  |  |  |
| May | 0.261 | -0.501 | 0.201 | -0.152 | 0.414 | -0.023 | 0.044 | 0.023 | -0.020 |
| June | 0.796 | -0.490 | 0.207 | 0.053 | 0.443 | 0.015 | 0.006 | 0.040 | -0.005 |
| July | 0.016 | -0.953 | -0.042 | 0.300 | -0.014 | 0.097 | -0.011 | 0.049 | 0.026 |
| August | -0.150 | -0.997 | -0.304 | 0.387 | 0.091 | 0.179 | -0.047 | 0.041 | 0.073 |
| September | -0.898 | -0.639 | -0.281 | 0.121 | -0.188 | 0.056 | -0.076 | 0.032 | 0.072 |
| October | -0.268 | 0.519 | -0.190 | -0.050 | -0.135 | 0.001 | -0.068 | -0.001 | 0.081 |
| November | -0.033 | 1.066 | -0.042 | -0.121 | -0.291 | -0.050 | -0.090 | -0.035 | 0.023 |
| December | 0.126 | 0.623 | -0.030 | 0.007 | -0.009 | -0.064 | -0.085 | -0.027 | 0.011 |
| 2022 | 1.459 | 1.041 | -0.061 | 0.071 | 0.581 | -0.063 | -0.028 | 0.040 | 0.058 |
| 2023 | 2.789 | 1.596 | -0.099 | 0.352 | 1.553 | -0.104 | -0.172 | -0.034 | 0.064 |

**Table S8. Ridge regression coefficients for VD, SE, CA, and IL, addressing multicollinearity in stepwise linear models. Coefficients are rounded to three decimal places.**

| **Response** | **VD** | **SE** | **CA** | **IL** |
| --- | --- | --- | --- | --- |
| Intercept | 0.559 | 0.170 | 0.111 | 0.082 |
| X_1_ | 0.000 | 0.000 | 0.000 | 0.000 |
| X_2_ | -0.001 | -0.001 | -0.001 | 0.000 |
| X_3_ | 0.000 | 0.000 | -0.001 | 0.000 |
| X_4_ | -0.003 | 0.001 | 0.000 | 0.000 |
| X_5_ | 0.000 | 0.000 | 0.000 | 0.000 |
| X_6_ | -0.001 | -0.001 | 0.000 | 0.000 |
| X_7_ | 0.000 | 0.000 | 0.000 | 0.000 |
| X_8_ | -0.001 | 0.000 | 0.000 | 0.000 |
| January | 0.004 | 0.005 | -0.005 | 0.000 |
| February | -0.018 | -0.008 | -0.012 | 0.000 |
| March | -0.009 | -0.010 | -0.004 | 0.000 |
| April |  |  |  |  |
| May | 0.009 | -0.006 | 0.003 | 0.000 |
| June | 0.025 | -0.006 | 0.012 | 0.000 |
| July | 0.009 | -0.002 | 0.005 | 0.000 |
| August | -0.019 | 0.016 | -0.006 | 0.000 |
| September | -0.031 | 0.014 | -0.011 | 0.000 |
| October | -0.019 | -0.001 | -0.004 | 0.000 |
| November | -0.009 | -0.002 | -0.001 | 0.000 |
| December | 0.012 | 0.008 | 0.004 | 0.000 |
| 2022 | 0.031 | 0.028 | 0.002 | 0.000 |
| 2023 | 0.033 | -0.004 | 0.020 | 0.000 |
