# Supplementary figures and images for: Environmental drivers of broiler carcass condemnation in humid subtropical regions: an exploratory study on the association of lagged climatic effects
Source: Trop Anim Health Prod. 2026 May 20;58(5):279. doi: 10.1007/s11250-026-05081-y (PMC13190353; doi:10.1007/s11250-026-05081-y)

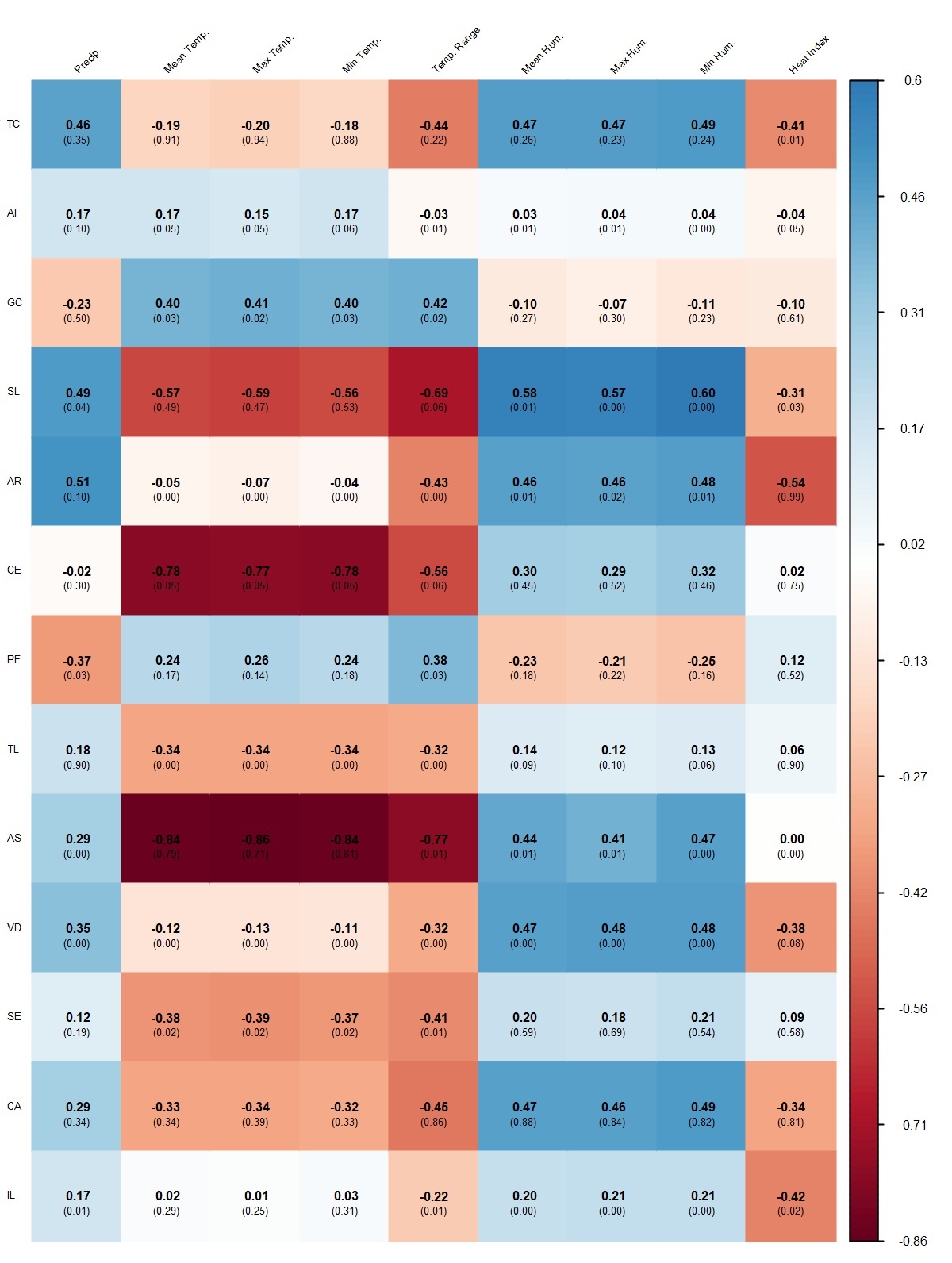

Supplement: Supplementary file 2 — Supplementary Material 2: Fig. S1: Correlation heatmap between poultry condemnation causes and climatic variables with lag of 1 month. Each cell shows the Spearman rank correlation coefficient with the corresponding p-value. P-values < 0.05 indicate statistically significant correlation. [file 11250_2026_5081_MOESM2_ESM.jpg]

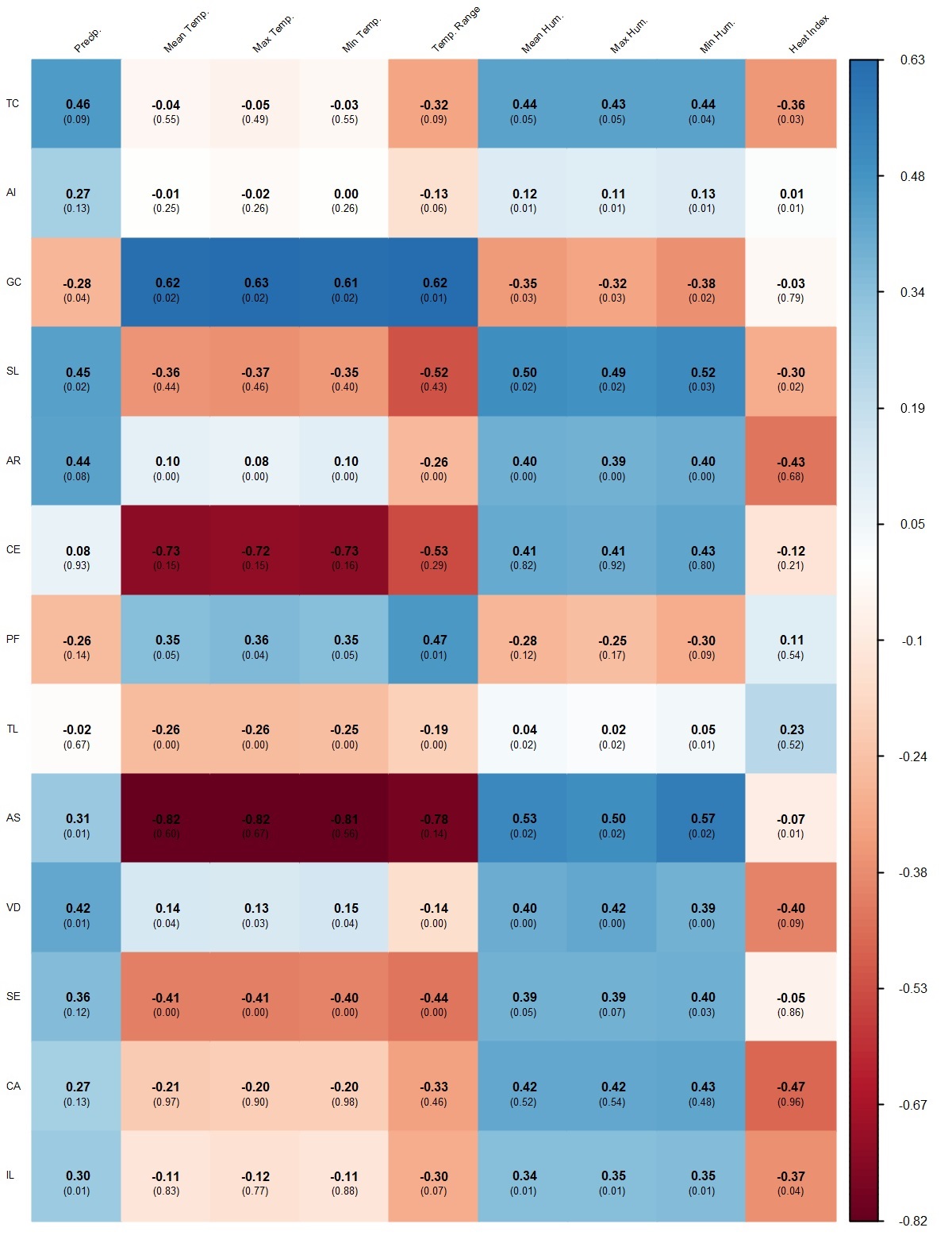

Supplement: Supplementary file 3 — Supplementary Material 3: Fig. S2: Correlation heatmap between poultry condemnation causes and climatic variables with lag of 2 months. Each cell shows the Spearman rank correlation coefficient with the corresponding p-value. P-values < 0.05 indicate statistically significant correlation [file 11250_2026_5081_MOESM3_ESM.jpg]

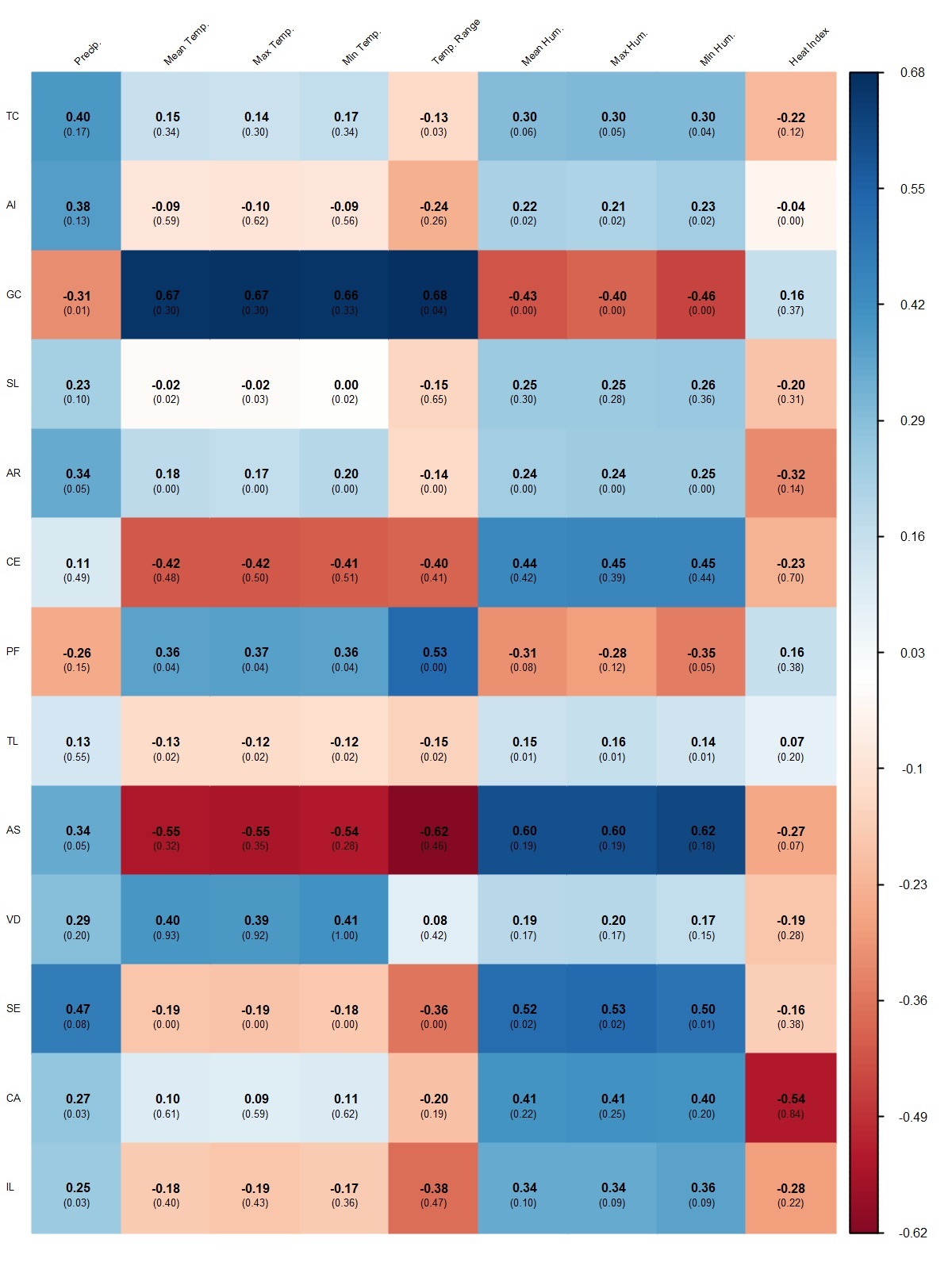

Supplement: Supplementary file 4 — Supplementary Material 4: Fig. S3: Correlation heatmap between poultry condemnation causes and climatic variables with lag of 3 months. Each cell shows the Spearman rank correlation coefficient with the corresponding p-value. P-values < 0.05 indicate statistically significant correlation. [file 11250_2026_5081_MOESM4_ESM.jpg]
